# Effects of Oral Glutamine Supplementation, Birthweight and Age on Colonic Morphology and Microbiome Development in Male Suckling Piglets

**DOI:** 10.3390/microorganisms10101899

**Published:** 2022-09-25

**Authors:** Johannes Schulze Holthausen, Johannes Schregel, Miriama Sciascia, Zeyang Li, Armin Tuchscherer, Wilfried Vahjen, Cornelia C. Metges, Jürgen Zentek

**Affiliations:** 1 Institute of Animal Nutrition, Department of Veterinary Medicine, Freie Universität Berlin, 14195 Berlin, Germany; 2 Research Institute for Farm Animal Biology (FBN), Institute of Nutritional Physiology, 18196 Dummerstorf, Germany; 3 Research Institute for Farm Animal Biology (FBN), Institute for Genetic and Biometry, 18196 Dummerstorf, Germany

**Keywords:** glutamine, colon, suckling piglets, low birthweight, intestinal morphometry, microbiota, bacterial metabolites

## Abstract

Mortality, impaired development and metabolic dysfunctions of suckling low-birthweight piglets may be influenced by modulating the intestinal microbiome through glutamine supplementation. Therefore, this study examined whether glutamine supplementation may affect the colonic development and microbiome composition of male low- and normal-birthweight piglets at 5 and 12 days of age. Suckling piglets were supplemented orally with glutamine or alanine. Colonic digesta samples were obtained for 16S rDNA sequencing, determination of bacterial metabolites and histomorphological tissue analyses. Glutamine-supplemented piglets had lower concentrations of cadaverine and spermidine in the colonic digesta (*p* < 0.05) and a higher number of CD3^+^ colonic intraepithelial lymphocytes compared to alanine-supplemented piglets (*p* < 0.05). Low-birthweight piglets were characterised by a lower relative abundance of *Firmicutes*, the genera *Negativibacillus* and *Faecalibacterium* and a higher abundance of *Alistipes* (*p* < 0.05). Concentrations of cadaverine and total biogenic amines (*p* < 0.05) and CD3^+^ intraepithelial lymphocytes (*p* < 0.05) were lower in low- compared with normal-birthweight piglets. In comparison to the factor age, glutamine supplementation and birthweight were associated with minor changes in microbial and histological characteristics of the colon, indicating that ontogenetic factors play a more important role in intestinal development.

## 1. Introduction

After birth, the neonatal piglet must adapt to a nonsterile environment and transition from uterine nutrition to colostrum and milk. This transition initiates the rapid development towards a maturing digestive and immune system. By suckling milk, piglets are provided with essential nutrients, such as lactose and proteins, as well as immunoglobulins and oligosaccharides [1]. Low-birthweight piglets (LBW), often born to sows with a high reproductive performance, have a higher risk of neonatal mortality and digestive disease, lower body weight (BW) gain [2] and impaired gastrointestinal (GIT) development [3].

There is increasing evidence that host–microbiota interactions are associated with nutrient uptake and metabolism, development of host immune functions and disease disposition [4]. It is known that the GIT microbiota are unstable in the first days of life [5] and influenced by the maternal and solid diet [6], as well as by the environment [7]. Neonates with intestinal microbial dysbiosis may be more susceptible to intestinal diseases [8]. The majority of studies investigating the development of GIT microbiota in newborn piglets report data from normal-birthweight (NBW) piglets [5,9,10], whereas few studies have looked more closely at the development of the colonic microbiota of LBW piglets [11]. Studies in neonatal intrauterine-growth-restricted piglets show that mucosa-associated bacterial colonisation was increased compared to NBW piglets [12,13]. Early age is characterised by a rapidly changing colonic [11] and faecal [14] microbiome. Microbial metabolites are considered as important factors for the microbiota–host cross-talk [10], impacting many physiological and immunological traits of the host. Short-chain fatty acids (SCFAs) and lactate contribute to meeting the energy needs in pigs, but also have an important signalling function [15]. SCFAs and biogenic amines, which control cell metabolism and may have neuromodulatory effects in animals [16], are considered important bacterial metabolites with physiological and immune-modulating functions [10]. Increasingly, data are available, showing that the mechanisms involve complex signalling systems and molecular cascades [15].

In sow milk, Gln and glutamate are highly concentrated peptide-bound amino acids. The free Gln concentration increases during lactation [17]. Glutamine is an important energy source for enterocytes of neonatal piglets [18]. Studies investigating the effect of enteral Gln on improving GIT development have mainly focused on weaned piglets, indicating its importance in numerous metabolic processes essential for the morphological development and function of the small intestine (SI) [19]. In addition, Gln has been shown to affect the bacteria of the SI and their AA utilisation pattern [20]. In suckling piglets, little is known about the effect of Gln supplementation on the colonic microbiome and important fermentation products such as SCFAs and biogenic amines.

Therefore, this study used a pig model with different birthweights (BiWs; LBW vs. NBW) and oral AA supplementation (Gln vs. Ala) across two different age groups (5 and 12 days old) to investigate their potential impact on colon development, the colon microbiome and targeted metabolites. Alanine was used as the control supplementation to balance for the nitrogen content of Gln supplementation [19,21,22,23].

## 2. Materials and Methods

### 2.1. Animals, Experimental Design and Sample Collection

All experimental procedures were performed according to the German Animal Welfare Act following Directive 2010/63/EU and were approved by the State Office for Agriculture, Food Safety and Fisheries, Mecklenburg-Vorpommern, Germany (permission no. 7221.3-1-026/16). German Landrace gilts were kept at the Research Institute for Farm Animal Biology. A detailed description of the experiment was published previously [17]. To remove sex-specific effects, only male piglets were chosen. In brief, LBW (0.8–1.2 kg; *n* = 48; with BiW below the lowest BiW quartile of the experimental pig farm) and NBW (1.4–1.8 kg; *n* = 48; with BiW reflecting the middle 50^th^ percentile of the BiW of piglets born on the experimental pig farm) male littermate piglets born to gilts were observed until 5 or 12 days (d) of age. Within 24 h post-farrowing, litters were standardised to 12 piglets, and the LBW and NBW piglets were assigned to either Gln (1 g/kg BW/d; *n* = 48) or isonitrogenous Alanine (Ala, 1.22 g/kg BW/d; *n* = 48) supplementation groups (Supp), with up to three piglet pairs per sow being involved in the study. In a three-factorial design (Supp, BiW, Age), 4 experimental groups (Gln-LBW, Gln-NBW, Ala-LBW, Ala-NBW: *n* = 24/age-group) were investigated at 5 or 12 d of age. The supplementation of Gln and Ala was performed as described [17].

Two hours (h) before sampling, each piglet received 33% of the respective daily AA supplement and 6 mL of milk replacer (150 g/L water at 45 °C; 16.5 MJ metabolisable energy (ME)/kg, 20.5% crude protein, 10.0% crude fat, 0.2% crude fibre; Neopigg Rescuemilk 2.0, Provimi, The Netherlands).

Colonic tissue and digesta were sampled from the ascending colon. After collection, the digesta was snap-frozen in liquid nitrogen and then stored at −80 °C until subsequent analysis. A section of the sampled colonic tissue was rinsed with 0.9% physiological saline, and preserved in Roti-Histofix (4% paraformaldehyde, Histofix, Roth, Karlsruhe, Germany) for histological analysis.

### 2.2. Colonic Morphometry, Histochemistry and Immunohistochemistry

Histo-fixed colonic tissue samples were processed as previously described [24]. From paraffin blocks, 5 μm sections were cut with a sledge microtome (Type 1400, Leitz Wetzlar, Germany). The Alcian blue pH 2.5–periodic acid–Schiff (AB-PAS) staining method was used for morphometry and for the quantification of neutral, acidic and mixed mucin types [25]. Measurements were carried out using a Photomicroscope BX43F (Olympus, Tokyo, Japan) with an attached digital camera (Olympus DP72, Tokyo, Japan). Pictures were examined with cellSens imaging software (v. 1.4, Olympus). Ten well-orientated crypts were randomly chosen. Morphometric measurements included crypt depth (CD) (from the crypt mouth to the bottom of the crypts) and crypt area (CA) [26]. Mucins in goblet cells were differentiated by AB-PAS staining [26].

For quantification of IgA secreting cells, slides were boiled in sodium citrate buffer (pH 6.0) in a microwave. Endogenous peroxidase was inactivated at room temperature for 30 min with 1% aqueous hydrogen peroxide solution. Slides were incubated for 1 h in a humid chamber with PBS and 10% normal horse serum to block nonspecific antibody binding. Sections were incubated overnight at 4 °C with goat anti-porcine IgA 1:4000 antibody (NB724, Novus Biologicals, Abingdon, UK), washed and incubated for 1 h with biotinylated horse anti-goat IgG 1:500 (Cat. NO: BA-9500, Vector Laboratories), treated with ABC complex (Vectastain Elite ABC peroxidase Kit, Standard, Vector Laboratories) and a DAB Substrate Kit (Vector Laboratories) [27]. Isotype control was produced with a nonspecific antibody (goat IgG, AB-108-c, R&D Systems). To quantify IgA-positive stained cells, 3 areas of lamina propria on each section were chosen [28]. The areas were delineated with cellSens imaging software (v. 1.4, Olympus), ignoring the epithelium, large blood vessels and artefacts. Positive stained cells were counted and expressed per 10,000 μm^2^ of lamina propria tissue [29].

The analysis of CD3^+^ intraepithelial lymphocytes (IELs) was performed as described previously [30], and the number of CD3^+^ IELs in the lamina propria next to the crypts was evaluated. Slides were heated for 30 min in boiling citrate buffer using a microwave. Slides were then cooled and incubated with a primary antibody PPT3 (mouse anti-porcine CD3 epsilon, CAT NO 4510-01, Southern Biotech) and an isotype control with a nonspecific antibody (mouse IgG, CAT NO 0102-01, Southern Biotech). The visualisation was achieved with the mouse and rabbit Specific HRP/DAB IHC Detection Kit (ab236466, ABCAM), and the secondary antibody was visualised with horseradish peroxidase (HRP)-labelled micropolymer (goat anti-rabbit HRP Conjugate, 58009 ABCAM) [31]. A double-blinded quantification of CD3-positive IELs was performed in well-orientated complete crypts (two slices per animal, ten crypts per slice). The CD3^+^ IELs were expressed per 100 enterocytes and CD3^+^ in the lamina propria per 10,000 μm^2^.

### 2.3. Chemical Analyses

Colon digesta SCFA and biogenic amines were quantified as described previously [32,33]. Briefly, SCFA analysis of digesta was performed by acidifying the samples with oxalic acid, followed by centrifugation for 3 min at 14,000 g and adding the internal standard (caproic acid). A gas chromatograph (Agilent Technologies 6890N, autosampler G2614A and injection tower G2613A; Network GC Systems, Böblingen, Germany) was used. Ion exchange chromatography was performed with a Biochrom 30 Amino Acid Analyzer (Biochrom) to analyse biogenic amines (putrescine, cadaverine, spermidine, spermine, propylamine, tyramine). Trichloroacetic acid (10%) was added to the digesta samples. After homogenisation and filtering (0.2 µm pore size), samples (25 µL) were injected onto a 10 cm polyamine ion-exchange column (Laborservice Onken GmbH, Gründau, Germany). The eluent was sodium citrate buffer (pH 7.2). Amines were quantified after post-column ninhydrin derivatisation by photometric detection at 570 nm [33].

### 2.4. DNA Extraction and 16S rDNA Sequencing

Bacterial genomic DNA was extracted from 250 mg digesta using a commercial kit, NucleoSpin Tissue Mini Kit for DNA from cells and tissue (NucleoSpin, Macherey & Nagel, Düren, Germany) according to the manufacturer’s instructions with the following exceptions: bead beating of 250 mg digesta in 1 mL of pre-lysis solution was carried out on a FastPrep-24™ 5G homogeniser (MP Biomedicals, LLC, Santa Ana, CA, USA) at a speed of 6 m/s for 10 min (4 times 5 × 30 s and 15 s cooling pause); Proteinase K treatment lasted for 30 min at 56 °C. The following steps were performed as described by the manufacturer, but the volume of the elution buffer was doubled to increase DNA yield. According to the manufacturer’s instructions, DNA concentration was determined using Promega QuantiFluor^®^ dsDNA System (Promega, Corporation, Madison, Wisconsin, USA). DNA extracts were subjected to amplicon sequencing using an Illumina NextSeq500 sequencer (LGC, Berlin, Germany) with 150 bp-paired reads using 16S rDNA primers 341f and 785r. Demultiplexing was achieved with Illumina bcl2fastq (v. 2.17.1.14); paired reads were combined with BBMerge (v. 34.48).

### 2.5. Data Evaluation and Statistical Analysis

A multivariate approach was used for statistical analyses of histological data, biogenic amines and SCFAs. Linear mixed model analysis was conducted using the ANOVA procedure of the IBM SPSS Statistics software Version 25 (IBM, Chicago, Illinois, USA). The three fixed-effects Supp (Ala, Gln), BiW (LBW, NBW) and Age (5 d and 12 d) and their interactions were tested, and the Tukey test was used for groupwise comparisons. Means and their standard errors are shown. Differences were considered statistically significant at *p* < 0.05 and as trends at *p* ≤ 0.1.

The 16S-rDNA sequences were analysed using the QIIME2 pipeline [34] and the SILVA SSU database [35]. Quality control and determination of sequence counts were performed using the DADA2 database software [36]. Further details were previously described [37]. The bacterial alpha-diversity measures Richness, Shannon Index and Evenness were calculated from ASV-level data. The Kruskal–Wallis test was used to test the effects of the main factors Supp, BiW and Age and their interactions on the bacterial abundance in the colon. A level of 95% was deemed as significantly different. Principal component analysis (PCA) and hierarchical clustering, using *Clustvis* [38], were used to visualise Supp, BiW and Age differences (Figure S1).

## 3. Results

### 3.1. Morphology of the Colon and Frequency of Goblet Cells, Intraepithelial Lymphocytes and IgA-Positive Cells

Colon tissue from Gln-supplemented piglets had a higher number of CD3^+^ IELs (*p =* 0.028) and showed a trend for an increase in the lamina propria (*p* = 0.054) (Table 1). A higher number of CD3^+^ IELs was observed in NBW compared to LBW piglets (*p =* 0.047).

**Figure 1:**
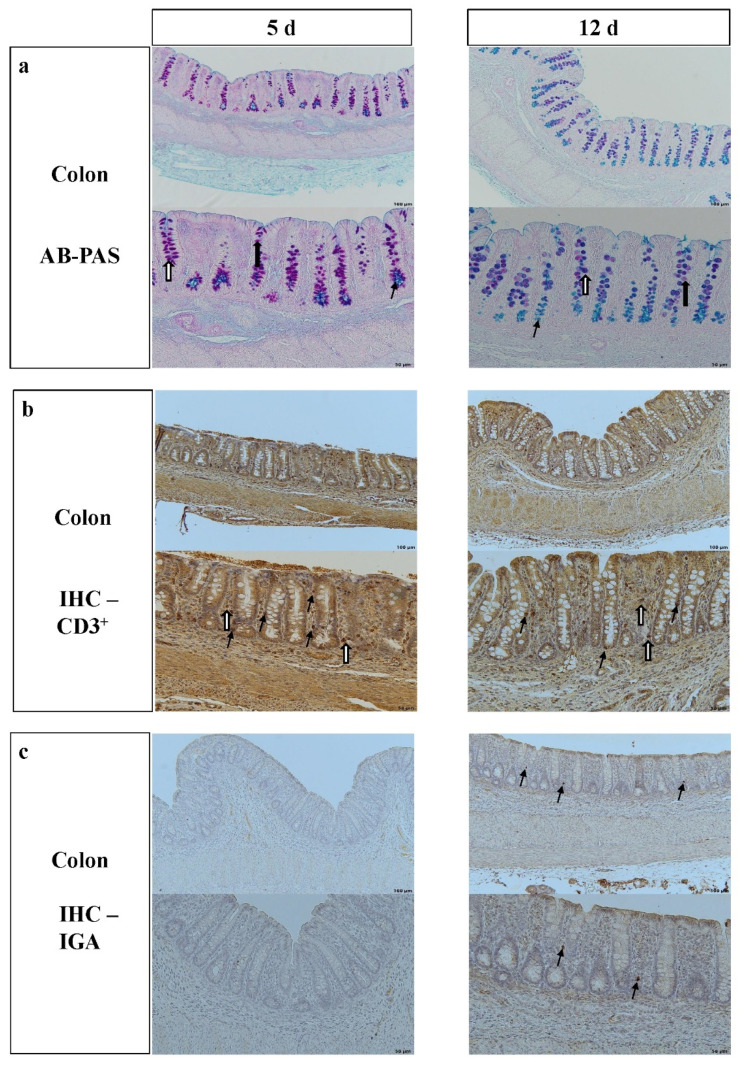
Histomorphology and immunohistochemistry of the colon of 5 d- and 12 d-old male suckling piglets. (**a**) Alcian blue pH 2.5–periodic acid–Schiff-stained colonic tissue with stained goblet cells, with different arrows indicating goblet cells containing different mucins, white arrow with black border = acidic mucins, black arrow = neutral mucins, white arrow = mixed mucins, 100× (upper pictures), 400× magnification (lower pictures); (**b**) IHC of CD3, with black arrows indicating positive stained intraepithelial CD3+ cells in colon, white arrows indicating positive stained CD3+ cells in lamina propria 100× (upper pictures), 400× (lower pictures) magnification; (**c**) IHC of IgA-positive stained cells in lamina propria, no IgA-positive cells detected at day 5, with arrows indicating IgA-positive cells, 100× and 400× magnification.

In piglets that were 12 d-old, CD and CA and the number of CD3^+^ IELs and CD3^+^ lymphocytes in the lamina propria (*p* < 0.001) were higher than at 5 d. However, the total numbers of goblet cells (*p* = 0.001) with neutral (*p* = 0.006) or mixed mucins (*p* < 0.001) were lower at 12 compared to 5 d of age. IgA-positive cells were absent in the colonic lamina propria in 5 d-old piglets, and were detected in all piglets at 12 d (Table 1, Figure 1)

The interactions of Supp × Age and BiW × Age were associated with changes in CD (*p =* 0.001; *p* < 0.001) and CA (*p =* 0.010; *p* = 0.002), respectively, and the interaction of Supp × BiW × Age was associated with changes in CD (*p =* 0.026), CA (*p =* 0.008) and CD3^+^ IELs *(p =* 0.043) and a trend for the number of mixed mucins (*p = 0.080*) (Tables S1–S3).

### 3.2. Bacterial Metabolites in the Colon Digesta

Glutamine-supplemented piglets had lower concentrations of cadaverine (*p* = 0.036) and spermidine (*p* = 0.020) and tended to have lower tyramine concentrations (*p* = 0.087) in the colonic digesta compared to Ala piglets (Table 2).

Normal-birthweight piglets had higher concentrations of cadaverine (*p* = 0.026) and total biogenic amines (*p* = 0.011) and a trend for increased concentrations of tyramine (*p* = 0.057) compared to LBW piglets (Table 2).

At 12 d, colonic digesta had higher tyramine (*p* = 0.019), putrescine (*p* = 0.018) and total biogenic amines (*p* < 0.001) and lower spermidine (*p* < 0.001) concentrations than at 5 d (Table 2).

The interaction of Supp × BiW showed a trend towards an effect on concentrations of propionic acid (*p* = 0.071), total SCFA (*p* = 0.074) (Table 3) and concentrations of cadaverine (*p* = 0.023), tyramine (*p* = 0.053), spermidine (*p* = 0.099) and total biogenic amines (*p* = 0.061).

The interaction of Supp × Age showed two trends, BiW × Age, with one significant effect and two trends and Supp × BiW × Age, with one significant effect and one trend for an effect on the concentration of biogenic amines (Table 2 and Table S5). The interaction of Supp × BiW × Age tended to affect the concentration of butyric acid (*p* = 0.090) (Table 3 and Table S6).

### 3.3. Impact of AA Supplementation on the Colonic Microbiota

Trends for lower relative abundances of *Planctomycetes* on the phylum level (Table 4) and on the order level and a trend for lower abundances of an unknown *Firmicutes* were detected in colonic digesta of Gln- compared to Ala-supplemented piglets (*p* = 0.054) (Table S7).

At the genus level, the relative abundances of *Phascolarctobacterium* (*p* = 0.086) and *Peptococcus* (*p* = 0.081) showed a trend of being higher (Table S8), and relative abundances of several unknown genera from the families of *Clostridiaceae 1* (*p* = 0.091), *Carnobacteriaceae* (*p* = 0.055) and *Streptococcaceae* (*p* = 0.053) tended to be lower in colonic digesta of Gln- compared to Ala-supplemented piglets.

### 3.4. Impact of Birthweight on the Colon Microbiota

Low-birthweight piglets were characterised by a lower abundance of the phylum Firmicutes (*p* = 0.049) and showed a trend for a decrease in the phylum *Lentisphaerae* (*p =* 0.080) compared to NBW piglets (Table 4). The order *Actinomycetales* tended to be increased in LBW compared to NBW piglets (*p* = 0.062) (Table S7).

On the genus level, LBW piglets had a higher relative abundance of *Alistipes* (*p* = 0.043) and a trend for slightly higher relative abundances of *Peptostreptococcus* (*p* = 0.087), *Mannheimia* (*p* = 0.075) and unknown *Desulfovibrionaceae* (*p* = 0.095) compared to NBW piglets (Table S8). In comparison to LBW piglets, the relative abundance of *Negativibacillus* (*p* = 0.020) and *Faecalibacterium* (*p =* 0.039) was higher and showed a trend for a slightly higher abundance of the genera *Dorea* (*p =* 0.066) and unknown *Prevotellaceae* (*p =* 0.063) in the colon digesta of NBW piglets.

### 3.5. Impact of Age on the Colon Microbiota

At the level of the phyla, a lower relative abundance of Verrucomicrobia (*p* = 0.002), Spirochaetes (*p* = 0.003), Tenericutes (*p* = 0.002), Epsilonbactereota (*p* < 0.001) and Kritimatiellaeota (*p* = 0.021) and a trend for slightly higher abundances of Bacteriodetes (*p* = 0.052) were detected in the colon digesta of piglets at 12 d compared to 5 d of age (Table 4).

At the level of bacterial order, the abundance of *Victivallales* (*p* = 0.037) and *Coriobacteriales* (*p* = 0.003) was lower at 5 than at 12 d (Table S7). *Lactobacillales* (*p* = 0.063), and *Bacteroidales* (*p* = 0.052) showed a trend for a higher abundance at 5 compared to 12 d. The relative abundance of unknown *WPS-2* (*p* = 0.002), *Desulfovibronionales* (*p* = 0.002), *Betaproteobacteriales* (*p* = 0.002), *Corynebacteriales* (*p* = 0.005) and *Campylobacterales* (*p* = 0.021) was lower at 5 than at 12 d, and a similar trend was found for *Spirochaetales* (*p* = 0.054), *Mollicutes* RF39 (*p* = 0.058) and *Micrococcales* (*p* = 0.092).

At the genus level, the relative abundances of 28 genera were higher (*p* < 0.05), and 8 tended to be higher (*p* < 0.1) in the colon digesta of 12 d- compared to 5 d-old piglets. Furthermore, the relative abundances of 26 genera were lower (*p* < 0.05), and another 8 genera tended to be lower (*p* < 0.1) in the colon digesta of 12 d- compared to 5 d-old piglets (Table S8). Of the dominating genera with a mean abundance > 1%, unknown *Muribaculaceae* (*p =* 0.001), unknown *Lachnospiraceae* (*p =* 0.013), *Lachnoclostridium* (*p =* 0.022) and *Parabacteroides* (*p* < 0.001) were lower at 12 d than 5 d, and the relative abundance of *Fusobacterium* (*p =* 0.052) and *Prevotellaceae* NK3B31 groups (*p* = 0.091) showed a trend for lower values at 12 d than at 5 d. The genera *Rombutsia* (*p* = 0.010), *Ruminococcaceae* UCG-002 (*p* = 0.004), *Ruminococcaceae* UCG-005 (*p* < 0.001), *Alloprevotella* (*p =* 0.024), *Christensenelllaceae* R-7 group (*p* < 0.001) and unknown F082 (*p* < 0.001) showed an age-dependent increase in colon digesta from 5 to 12 d of age (Table S8).

### 3.6. Interaction of Supplementation, Birthweight and Age Effects on Bacterial Phyla, Order and Genera

The interaction of Supp × BiW (*p* = 0.009) and Supp × BiW × Age (*p* = 0.032) influenced the abundance of *Firmicutes*. Several other significant and trends for interactions for bacterial phyla with a relative abundance < 1%, mainly influenced by the factor Age, are shown in Table 4.

The interaction of Supp × BiW influenced the relative abundance of *Bradymondales* (*p* = 0.024) and showed a trend for an influence on the relative abundance of *WCHB1*-41 (*p =* 0.096). In total, seven significant and two trends for the interaction of Supp × BiW, six significant and one trend for the interaction of BiW × Age and three trends for the interaction of Supp × BiW × Age of bacterial abundances < 1% on the order level are shown in Tables S7 and S11.

The interaction of Supp × BiW affected the relative abundances of unknown *Bradymondales* (*p* = 0.024), unknown *Prevotellacae* (*p* = 0.049), *Alistipes* (*p* = 0.030), *Staphylococcus* (*p* = 0.028) and *CAG*-873 (*p* = 0.015) in colonic digesta. Moderate interactions of Supp × BiW and effects of the other interactions (Supp × Age, BiW × Age; Supp × BiW × Age), again mainly influenced by the factor Age, were found and are shown in Tables S8 and S12.

### 3.7. Quantitative Analysis, Ecological Indices and Principal Component Analysis of the Colonic Microbiota

**Figure 2:**
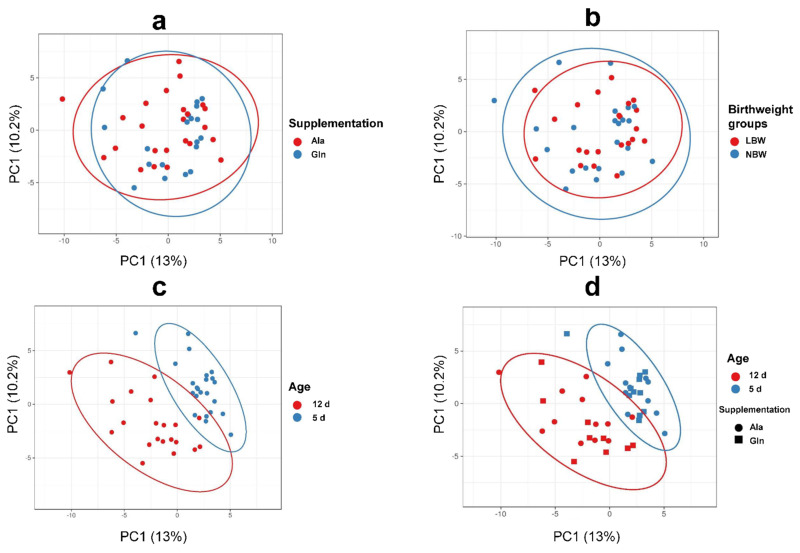
Principal component analysis (PCA). Principal component analysis (PCA) showing the effect of (**a**) supplementation; (**b**) birthweight; (**c**) age; (**d**) age and supplementation × birth weight on bacterial genus level. Ala = Alanine, Gln = Glutamine, LBW = low birthweight, NBW = normal birthweight. PCA was performed with Clustvis.

The main abundant phyla in the colon digesta of male suckling piglets were *Firmicutes* and *Bacteroidetes,* followed by *Fusobacteria* and *Proteobacteria* (Table 4). On the order level, *Clostridiales, Lactobacillales* and *Bacteroidales* were most abundant (Table S1). Regardless of Supp, BiW and Age, the dominating bacterial genera were *Lactobacillus* and *Clostridium sensu stricto* 1 (Table S2). Neither Supp nor BiW or Age affected Richness, Evenness or Shannon Indices (Table 4). Principal component analysis of all bacterial genera revealed no separation between Supp or BiW (Figure 2a,b); however, Age did, with 5 d-old piglets clustered separately from 12 d-old piglets (Figure 2c,d).

### 3.8. Correlations between Microbiota and Bacterial Metabolites in Colon Digesta

**Figure 3:**
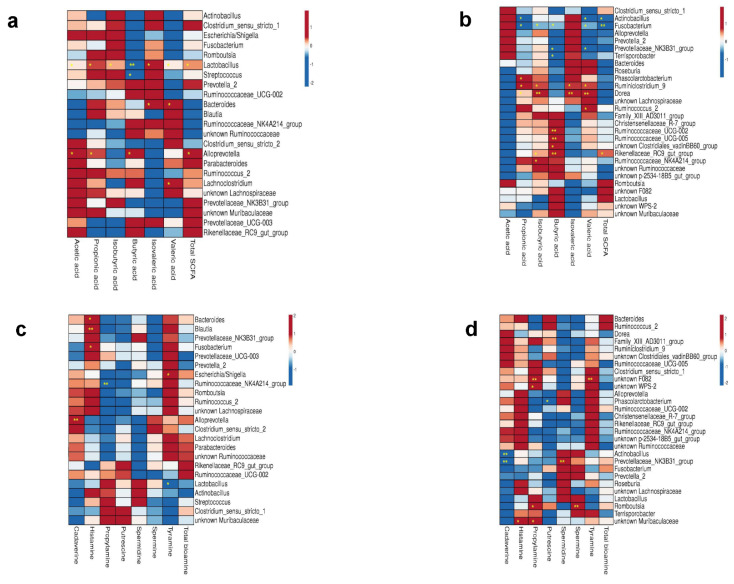
Concentration of bacterial metabolites and their correlation with bacteria having an abundance > 0.5%. The data are presented as mean values, *n* = 5 for SCFAs between groups; n = 2 for biogenic amines at 5 d of age and *n* = 4 at 12 d of age; the colours range from blue (negative correlation) to red (positive correlation). Significant correlations are marked by ** *p* < 0.01 and * *p* < 0.05; (**a**) Spearman’s correlation between colonic microbiota with a general abundance > 0.5% and SCFAs at day 5; (**b**) Spearman’s correlation between colonic microbiota with a general abundance > 0.5% and SCFAs at day 12; (**c**) Spearman’s correlation between colonic microbiota with a general abundance > 0.5% and biogenic amines at day 5; (**d**) Spearman’s correlation between colonic microbiota with a general abundance > 0.5% and biogenic amines at day 12. The colours range from blue (negative correlation) to red (positive correlation). Significant correlations are marked by ** *p* < 0.01 and * *p* < 0.05.

A link between the colonic metabolites and the major genera was investigated by Spearman correlation. The Ala and Gln Supp groups were taken together because the bacterial abundance and the metabolite concentrations did not show major significant changes between groups. Figure 3a,b show the correlations between SCFAs and major bacteria genera (mean abundance > 0.5%) in the colon digesta of 5 d- and 12 d-old piglets. Total SCFAs and acetic and propionic acid positively correlated with the abundance of *Lactobacillus* and *Alloprevotella* at 5 d, respectively (*p* < 0.05). Butyric acid showed a significant negative correlation with the abundance of *Lactobacillus* and *Streptococcus* at 5 d, whereas the genus *Alloprevotella showed a* positive correlation (*p* < 0.05). At 12 d of age, total SCFAs negatively correlated with the abundance of *Fusobacterium* and *Actinobacillus,* whereas they positively correlated with the genera *Rikenellaceae RC9 gut group*. Propionic acid also showed a negative correlation with *Fusobacterium* and *Actinobacillus* and a positive correlation with the genera *Phascolarctobacterium*, *Ruminiclostridium 9* and *Dorea*. Butyric acid was negatively correlated with the abundance of *Fusobacterium, Prevotellaceae* NK3B31 group and *Terrisporobacter* and positively correlated with the *Rikenellaceae* RC9 gut group, *Ruminococcaceae* UCG 002/UCG 005 and unknown *Clostridiales vadin* BB60 group. Few correlations between the biogenic amines and major bacteria genera (mean abundance > 0.5%) at 5 d (Figure 3c) and 12 d (Figure 3d) could be detected. We found the most positive correlations between different bacterial genera and histamine at 5 d of age. At 12 d of age, most positive correlations between different bacterial genera and propylamine were detected. Cadaverine showed a negative correlation with the genera *Actinobacillus* and *Prevotellaceae* NK3B31 group in 12 d-old piglets.

## 4. Discussion

The objective of the study was to follow up on the results of a previous study in which Gln was administered to neonatal suckling piglets. That study showed improved growth, milk intake and lipid metabolism in LBW pigs, and associations with AA metabolism in NBW piglets [17]. Therefore, in the present study, we further investigated the impact of Gln, BiW and age on the colonic microbiota composition, microbial metabolites, mucosal morphology and immune cell density. We hypothesised that Gln supplementation from 1 to 12 d of age is associated with changes in the intestinal microbiota and microbial metabolites, also impacting the lower intestinal tract. Our results show some interesting effects in Gln-supplemented piglets. However, age had the most profound influence.

To the best of our knowledge, this is the first study investigating the effects of Gln supplementation in the ascending colon of piglets during the early suckling phase. Few studies with suckling piglets have investigated histological and immunologic parameters, and the microbiota and the metabolites of the colon [9,10,11]. However, the majority of studies investigated Gln and its relation to the development and function of the SI after weaning [19,23], while this was rarely explored in suckling piglets [21,39]. A possible downstream transfer of beneficial effects of Gln supplementation from proximal gut segments to the large intestine has not been characterised.

### 4.1. Effects of Gln Supplementation

Glutamine is an important energy source for immune cells in the GIT of piglets [23]. Within the adaptive immune system, T lymphocytes in the intestinal epithelium play a significant role in the gut barrier function, defence and tolerance mechanisms. Intraepithelial lymphocytes have a primary function for maintaining gut health in early life. They are one of the first cells with an immunological function protecting the intestinal epithelium [40]. Therefore, a higher number of CD3^+^ IELs in Gln- compared to Ala-supplemented piglets might indicate a more maturate intestinal immune system. The observed difference in CD3^+^ IELs could be related to T-cell-dependent pathways via a direct effect of Gln supplementation on the intestinal microbiota in more proximal gut segments [41]. An explanation for the absence of effects of Gln supplementation on most of the examined morphological colonic parameters could be due to the metabolism and absorption of the supplemented Gln in proximal parts of the SI with only minor changes in AA concentrations in jejunal digesta and tissue [42] and possibly only little carry-over effects of proximal metabolic products into distal gut segments [43].

In our study, colonic SCFA concentrations remained largely unaffected by Gln supplementation. The relative abundance of *Lactobacillus* spp. was quite similar between Gln- and Ala-supplemented suckling piglets. There was obviously no promotion of SCFA production by the presence of *Lactobacillus* spp., which may also be due to the fact that 5 d- or 12 d-old animals still show extreme fluctuations in the microbiome, which is not as stable as after weaning [10]. Biogenic amines are products of bacterial AA decarboxylation, whose biological importance has been increasingly recognised for both the microbiota and the intestinal tissue [44]. A lower pH is important for the AA decarboxylation activity, and *Lactobacillus* ssp. are mainly responsible for the synthesis of biogenic amines [45]. This is why decreased concentrations of cadaverine and spermidine in the Gln-supplemented piglets in our study might reflect different microbial abundances and microbial fermentation profiles in the colon or more proximal gut segments. Putrescine, spermidine and cadaverine influence gut maturation in weaned piglets [46]. Spermidine is believed to contribute to gut maturation in young piglets. Therefore, it could be assumed that a lower concentration of spermidine in the colon might indicate a more immature gut with reduced autophagic activity [46]. All in all, knowledge on the effects of intestinal biogenic amines in suckling piglets is scarce, but it is important to note that the function of biogenic amines is probably dependent on their concentration and the condition of the host [47].

Glutamine is extensively catabolised by bacteria in the SI of pigs and mice [22,43]. It was shown that 1% Gln supplementation in six week old mice had an influence on microbial composition in the jejunum and ileum, and activated proinflammatory processes through TLR4-nuclear factor κB (NF-κB), mitogen-activated protein kinases (MAPKs), and phosphoinositide-3 kinases (PI3Ks)/PI3K-protein kinase B (Akt) signalling pathways [43]. Even if Gln is mainly utilised in the SI, bacteria and metabolic products might reach the large intestine and influence the microbial composition and metabolic activity. Studies investigating effects of Gln or AA blends on the colonic microbiota have not been reported in suckling piglets. In weaned pigs, blends of Glu, Gln, glycine, arginine and N-acetylcysteine, added at 1% in the diet, increased *Lactobacillus* and *Bifidobacterium* spp. in mid colonic digesta [48]. In our study, the microbial abundance in colonic digesta was only moderately affected by Gln supplementation, similar to findings in rats [43], rabbits [49] and in faecal samples of underweight infants [50]. Effects of AA supplementation might be influenced by individual microbial composition in the colon of weaned piglets [51]. The individual variability in microbial AA degradation in suckling piglets, on the other hand, is not known.

### 4.2. Effects of Birthweight

Intraepithelial lymphocytes are components of the gut-associated lymphoid tissue, and are a first line of defence against infection [40]. In the current study, we show that LBW was associated with a lower number of CD3+ IELs in the colon compared to NBW, possibly indicating a better adaptive immune response of NBW piglets. A connection between the number of IELs and BW has been described in other animal species. For example, it was shown in mice that IELs have an important function in promoting weight gain [52]. This is possibly associated with the protective function of IELs in the gut [40].

Dietary protein has been reported to be mainly responsible for the concentration of biogenic amines in the colon of pigs [53]. While branched-chain fatty acids are produced by the deamination of valine, leucine and isoleucine, amines are produced by the decarboxylation of different AAs [53]. The digestive system of piglets in the current study can be considered as rapidly developing. Higher milk intake and the rapidly changing intestinal microbiome might lead to higher total biogenic amine and cadaverine concentrations in the colonic digesta of NBW piglets. It is known that the diamine cadaverine is almost only synthesised by bacteria [54].

Normal-birthweight piglets have different abundances of bacteria in the faeces [14,55], ileum and colon [11] during suckling and weaning compared with LBW piglets. At 3 and 7 d after birth, it has been reported that LBW suckling piglets (0.75–0.95 kg BiW) have a lower faecal abundance of *Firmicutes* than NBW (1.35–1.55 kg BiW) [14]. The higher relative abundance of *Firmicutes* in NBW piglets observed in the current study might relate to similar findings in obese minipigs [56]. Thus, a higher BW in NBW compared to LBW piglets [17] could be associated with a higher abundance of *Firmicutes.* However, overall, the abundance of the major bacterial genera was similar between LBW and NBW piglets. Most changes have been reported to occur in the minor bacterial genera in the colon and faeces of suckling piglets [11,14,55], which is in line with the current study. The faecal microbiota of LBW piglets were characterised by a lower relative abundance of *Lactobacillus*, *Streptococcus* and *Faecalibacterium* spp. and a higher proportion of *Fusobacterium* spp. at 3 and 7 d of age [14]. Piglets with a low daily BW gain at the ages of 4, 8 and 14 d have been reported to have lower faecal abundances of *Lactobacillus*, unclassified *Prevotellaceae* and *Ruminococcaceae* UCG-005 [55]. To the best of our knowledge, there appears to be only one other study [11] comparing the colonic microbiota of LBW and NBW suckling piglets. In the current study, LBW piglets had lower abundances of colonic *Alistipes*, *Lachnospiraceae*, *Ruminococcaceae* and *Prevotellaceae* and *Faecalibacterium* spp. Piglets harbouring increased levels of *Faecalibacterium* in the GIT showed a lower risk for diarrhoea after weaning [57]. In humans, this genus is associated with a lower incidence of inflammatory bowel disease and colorectal cancer [58]. In contrast to previous studies [11,55], we did not observe lower microbial abundances of *Bacteroidetes*, *Bacteroides* and *Ruminococcaceae*, especially *Ruminococcaceae* UCG-005 in LBW piglets. This relative stability of the microbiota might explain the similar SCFA concentrations in the colonic digesta. Interestingly, we observed a higher relative abundance of the genus *Alistipes* in colon digesta of LBW compared to NBW piglets. Low birthweight is associated with increased body fat accumulation [59], hepatic lipid droplets, the rate of lipolysis in the liver [60] and the number of intramyocellular lipid droplets in juvenile pigs [61]. *Alistipes finegoldii* belonging to the *Bacteroidetes* phylum has been shown to be a resident in the human gut microbiome and is involved in lipid metabolism via the bacterial type II fatty acid biosynthesis system [62]. The genus *Alistipes* has been associated with increased porcine back fat mass, intramuscular fat accumulation [63] and lean body mass in pigs [64], while in humans, health-protective effects have been reported to be related to liver fibrosis, colitis, cancer and gut dysbiosis [65].

As described previously [55], only minor effects of BiW on the microbiota, bacterial metabolites and histomorphomometric traits may have been observed, due to the higher BiW of selected LBW piglets compared to other studies [11,12,13,14]. Since studies on the histomorphological development of the large intestine in suckling piglets are scarce, especially those comparing LBW and NBW piglets, it should be mentioned that in this and in previous studies, using intrauterine-growth-restricted (BiW < 1.15 kg) and NBW piglets (1.25–1.70 kg BiW), no major morphological differences in the colon were detected in piglets at 5 d of age or earlier [12,13]. In addition, the lack of differences observed between LBW and NBW piglets in the relative abundance of the colonic microbiota reported in this study may also explain the lack of differences in bacterial metabolites concentrations. A possible explanation could be that biogenic amines and SCFAs are primarily produced by the dominant (>1% relative abundance) bacteria [10], and all other bacteria probably do not contribute strongly to the production of metabolites. Another explanation could be that possible birthweight-dependent differences may have been reduced during postnatal development when milk intake was high [66]. Furthermore, if the piglets survive the first 3 days of life, and suckle enough sow milk including immunoglobulins, antibodies and milk oligosaccharides [67], they become less comparable to very low birthweight piglets, which have been shown to differ in intestinal development, gene expression and bacterial profiles compared to NBW piglets immediately after birth [3,12,13,23].

### 4.3. Comparison of Age Groups

In the present study, the CD and CA of the colon and numbers of CD3+ IELs, CD3+ lymphocytes in the lamina propria and the number of IgA-positive cells as markers of immunological development increased with age, indicating a highly dynamic gut development in suckling piglets. Comparable results were obtained in piglets from 1 to 42 d of life observing an increased CD and increased expression of Toll-like receptors 2 and 9, indicating a better immune protection against pathogens [10]. The increase in CD was also confirmed in 0 d- compared to 28 d-old pigs [68]. The increases in CD are associated with increases in the absorptive surface and mucosal mass and could be related to factors such as intake of sow’s faeces and spilled feed or the developing intestinal microbiota. The influence of the intestinal microbiota on the intestinal architecture has already been shown. For example, the SI of germfree mice had shorter crypts [69], and similar findings, such as reduced mitotic index and cell turnover rate in the intestinal epithelium of colon and ileum of rats, were reported [70]. A decrease in the abundance of goblet cells containing different mucins in colonic crypts could also be dependent on the interaction with luminal and mucosal gut bacteria and the changing immune system due to maturation as was assumed earlier [9]. Goblet cells provide mucins for the mucus layers found in the colon. The outer layer, mainly consisting of MUC2, is permeable to bacteria. The tightly adherent inner layer, including different mucins impermeable to bacteria, contributes to the strong barrier function in the colon [71]. Decreasing densities of goblet cells in our study from 5 to 12 d of age are in accordance with a previous study observing a decrease in goblet cell density from 0 to 7 days of age and an increase at 14 d of age [9]. Examination of mucin composition revealed a relatively constant number of goblet cells containing acidic mucins from 5 d- and 12 d-old piglets, mostly located in the bottom of the crypts. The location of goblet cells containing acidic mucins was also seen in colonic crypts of piglets after weaning [26]. The balance between the major commensal bacteria leads to colonic epithelial homeostasis due to their effect on mucus production [72]. *Bacteroides* spp. positively affect mucus production, reducing neutral and mixed goblet cells in piglets [72]. In addition, it could be assumed that different bacteria ferment mucins. Therefore, the general bacterial interaction could be responsible for the abundance of mucins [72].

A higher frequency of CD3+ cells might indicate a more mature immune system in 12 d- compared to 5 d-old piglets. It is known that an adequate density of CD3+ IELs sustains epithelial barrier function [73]. The lower number of neutral, mixed and total goblet cells at 12 d of life may indicate that other factors contribute to an efficient barrier function. Due to the increasing milk intake of piglets in the first two weeks of life, it could be that sow milk including immunoglobulins, antibodies and milk oligosaccharides could lead to adequate protection. Therefore, the described protecting effect of milk oligosaccharides on the intestine [67] might lead to lower mucus production as the intestinal barrier function of the gut is intact and improved. At this point, it is of interest that milk oligosaccharides have a similar structure to mucin glycans, possibly having similar tasks in barrier function next to the known immunomodulatory and microbial effects [67]. Therefore, the intestinal barrier function in the colon might be supported by maternal milk and the higher number of CD3+ IELs.

IgA-positive B cells function as part of the innate immune defence after migrating from the Peyer’s patches into the lamina propria [74]. Factors such as age [75], the composition of the microbiota [76] and diet [77] seem to influence the abundance of IgA-positive B cells. The lack of IgA-positive cells at 5 d of age is in line with previous studies [75]. The emergence of an active immune system in the colon at the second week of life was demonstrated in the current study by the detection of IgA-positive cells in the lamina propria.

The primary site of lactate production is the upper GIT, where it is mainly produced by *Lactobacillus* spp., having beneficial effects on gut health, while acetate, proprionate and butyrate are mainly produced by specific microbial communities in the colon [15]. Arnaud et al. [9] reported increases in colonic SCFAs in the early postnatal period (7 to 14 d of age), whereas Li et al. [11] (7 to 21 d of age) and Qi et al. [10] (7 to 14 d of age) did not observe age-dependent changes, which is in line with the current study.

Polyamines have been reported to have concentration-dependent protective effects [46], and it has been suggested that putrescine, spermidine and cadaverine have an influence on gut maturation in weaned piglets. In contrast to Qi et al. [10], we observed a lower spermidine concentration and a higher total biogenic amine concentration in colon digesta in 12 d- compared to 5 d-old piglets. A decrease in putrescine concentrations in colon digesta of 7 d- compared to 14 d-old piglets has been previously reported [10]. The lack of agreement between the studies could be due to creep feeding (not in the current study) or the physiologically decreasing protein content of sow milk during lactation [78]. Higher tyramine and total biogenic amine concentrations in the colonic content of piglets could be due to a higher intake of indigestible nutrients and immature digestive function. Tyramine and other biogenic amines are produced by gut microbiota degradation of AAs [44]. A decrease in tyramine was observed in piglets right after weaning [79], which could be related to lower or no feed intake. In this study, piglets consumed more nutrients with increasing age, which might have led to an increase in tyramine and other biogenic amines.

The core microbiota of the colon and faeces are the *Firmicutes* and *Bacteroidetes* [9,10,11,14,55], and we observed here that they are the most abundant microbial phyla in the colonic digesta of neonatal piglets. The abundance of the genus *Lactobacillus* did not change in the colon digesta between 5- and 12-day-old piglets. Most of the initially less abundant bacterial phyla increased in number with age in the colon digesta, in line with previous observations [10,55]. Unknown *Muribaculaceae*, the fourth most abundant genus, decreased in abundance from 5 to 12 d. The functional role is not yet clear, but *Muribaculaceae* can degrade carbohydrates, although lower abundances were observed in mice and rats fed carbohydrate-enriched diets [80]. The observed increased abundance in the *Ruminococcaceae* family with age has also been described in previous studies [11,14,55]. *Ruminococcacae* can ferment dietary fibre, produce SCFAs and are considered as a dominant part of the microbiota of the pig colon [55]. In our study, *Ruminococcacceae* abundance was positively correlated with butyric acid concentration. It is not known whether bacteria belonging to the *Ruminococcaceae* family are involved in the fermentation of milk oligosaccharides to produce SCFAs, as has been observed for *Lactobacillus* and *Bifidobacterium* [67]. However, unknown interactions with the other members of the colon microbiota might also increase the abundance of *Ruminococcaceae*. These findings might indicate the maturation of the intestinal microbiota and the immune system of neonatal piglets towards increasing protection of the intestine in the first weeks of life or the protective function of milk oligosaccharides against possible pathogens [67].

Changes in the diversity of the colonic microbiota were identified in the first week of life and after 21 d [10] or 28 d [9] of life in piglets with no major changes in-between. Additionally, the bacterial abundance depends on the location and type of the sample as well as on nutritional and environmental factors [43] Another explanation for minor differences in bacterial abundances of the colon between 5 d- and 12 d-old suckling piglets could be that the colonic microbial composition of suckling piglets during the first two weeks of life is more likely driven by the milk oligosaccharides in sow milk [67] or by the sow–piglet relationship [81].

## 5. Conclusions

Glutamine supplementation, compared to Ala, and birthweight had minor effects on colonic development, microbial composition and microbial metabolites in piglets, whereas most of the observed effects were age-dependent. Glutamine supplementation increased the number of CD3+ IELs in the colon as well as the concentration of some biogenic amines in the colonic digesta. Our data suggest the effects of Gln supplementation are less distinct in distal parts of the gastrointestinal tract in neonatal piglets.

## Figures and Tables

**Table 1:** Morphometric and immunohisto-morphometric measurements of the colon of 5- and 12-day-old male suckling piglets ^1^.

	Supp		BiW		Age			*p* Values ^5^						
Item	Gln	Ala	LBW	NBW	5 d	12 d	SEM	Supp	BiW	Age	Supp × BiW	Supp × Age	BiW × Age	Supp × BiW × Age
**Morphometry**														
CD, μm	237	236	234	238	210	263	2.13	0.730	0.197	**<0.001**	0.490	**0.001**	**<0.001**	**0.026**
CA, μm^2^	10,950	10,993	10,964	10,979	9389	12,554	144	0.842	0.947	**<0.001**	0.259	**0.010**	**0.002**	**0.008**
**AB—PAS staining of Goblet cells ^2^**														
Acid	21.9	24.7	22.7	23.8	21.7	24.8	1.07	0.196	0.609	0.145	0.234	0.684	0.244	0.681
Neutral	67.1	69.2	70.9	65.4	75.4	60.9	2.64	0.686	0.280	**0.006**	0.296	0.915	0.527	0.128
Mixed	84.1	87.1	89.2	82.0	96.4	74.8	2.60	0.513	0.130	**<0.001**	0.330	0.759	0.303	***0.080***
Total	173	181	183	171	194	161	5.25	0.430	0.248	**0.001**	0.213	0.911	0.578	0.130
**CD3^+^ lymphocytes ^3^**														
CD3^+^ IEL	1.97	1.76	1.77	1.96	1.15	2.60	0.07	**0.028**	**0.047**	**<0.001**	0.255	0.107	0.165	**0.043**
CD3^+^ lamina propria	8.70	8.28	8.83	8.65	5.90	11.2	0.18	***0.054***	0.109	**<0.001**	0.911	0.495	0.514	0.603
**IgA-positive cells in lamina propria ^4^**														
IgA-positive cells	8.17	8.71	8.04	8.83	n.d	8.44	0.54	0.627	0.479	n.a	0.911	n.a	n.a	n.a

^1^ Values are means, the SE of all groups is shown; *n* = 48/group. Colon samples were obtained 2 h after oral supplementation of Gln or Ala and fixed in Formalin. ^2^ Number of Alcian blue–periodic acid–Schiff (AB-PAS)-positive goblet cells per 1 mm basal membrane. ^3^ Number of CD3^+^-positive lymphocytes per 100 enterocytes and per 10 000 μm^2^ lamina propria next to the crypts. ^4^ Number of IgA-positive cells per 10,000 μm^2^ of lamina propria. ^5^ ANOVA *F* test; significant differences (*p <* 0.05) are marked in bold, trends (*p* < 0.1) are marked in italic and bold. Ala = Alanine; Acid = acidic mucins; BiW = birthweight; CA = crypt area; CD = crypt depth; Gln = Glutamine; IELs = intraepithelial lymphocytes; LBW = low birthweight; n.a = not available (no IgA-positive cells detectable at 5 d); NBW = normal birthweight; n.d = not detectable; Mixed = mixed neutral and acidic mucins; SE = standard error; Supp = supplementation group; Total = total number of AB-PAS-positive goblet cells.

**Table 2:** Concentrations of biogenic amines in the colon digesta of 5- and 12-day-old male suckling piglets ^1^.

	Supp		BiW		Age			*p* Values ^2^						
Item, µmol/g Wet Weight	Gln	Ala	LBW	NBW	5 d	12 d	SEM	Supp	BiW	Age	Supp × BiW	Supp × Age	BiW × Age	Supp × BiW × Age
Spermine	0.02	0.03	0.03	0.03	0.03	0.03	0.00	0.275	0.534	0.764	0.351	0.840	0.744	***0.076***
Cadaverine	0.05	0.26	0.04	0.27	0.04	0.27	0.07	**0.036**	**0.026**	0.253	**0.023**	0.163	0.189	0.258
Tyramine	0.06	0.17	0.05	0.17	0.05	0.17	0.05	***0.087***	***0.057***	**0.019**	***0.058***	0.152	***0.086***	**0.049**
Propylamine	0.06	0.03	0.04	0.05	0.04	0.05	0.01	0.111	0.676	0.198	0.931	***0.067***	0.672	0.712
Histamine	0.07	0.09	0.07	0.09	0.07	0.09	0.02	0.698	0.611	0.546	0.192	0.725	0.901	0.319
Spermidine	0.29	0.39	0.36	0.33	0.36	0.33	0.03	**0.020**	0.205	**<0.001**	***0.099***	0.485	**0.034**	0.427
Putrescine	0.58	0.46	0.46	0.58	0.46	0.58	0.07	0.152	0.208	**0.018**	0.470	***0.074***	0.189	0.819
Total biogenic amines	1.14	1.43	1.05	1.52	1.05	1.52	0.15	0.130	**0.011**	**<0.001**	***0.061***	0.816	***0.098***	0.188

^1^ Values are means, the SE of all groups is shown; *n* = 10 group (not enough colonic digesta of all piglets available for analyses). Colon digesta samples were obtained at 2 h after oral supplementation with Gln or Ala and milk replacer, and snap-frozen in liquid nitrogen. ^2^ ANOVA *F* test; significant differences (*p* < 0.05) are marked in bold, trends (*p* < 0.1) are marked in italic and bold. Ala = Alanine; BiW = birthweight; Gln = Glutamine; LBW = low birthweight; NBW = normal birthweight; SE = standard error; Supp = supplementation group.

**Table 3:** Short chain fatty acid concentrations in the colon digesta of 5- and 12-day-old male suckling piglets ^1^.

	Supp		BiW		Age			*p* Values ^2^						
Item, mmol/L	Gln	Ala	LBW	NBW	5 d	12 d	SEM	Supp	BiW	Age	Supp × BiW	Supp × Age	BiW × Age	Supp × BiW × Age
Acetic Acid	26.2	27.2	26.8	26.6	27.1	26.2	1.39	0.726	0.948	0.753	0.173	0.804	0.531	0.621
Propionic acid	7.94	9.49	8.19	9.24	8.07	9.36	0.83	0.359	0.532	0.445	***0.071***	0.737	0.893	0.222
Isobutyric acid	1.39	1.49	1.38	1.49	1.39	1.48	0.10	0.645	0.594	0.671	0.252	0.627	0.894	0.597
Butyric acid	3.00	3.60	3.03	3.57	2.86	3.73	0.39	0.436	0.487	0.263	0.101	0.520	0.870	***0.090***
Isovaleric acid	1.25	1.39	1.23	1.41	1.23	1.40	0.09	0.451	0.328	0.372	0.363	0.417	0.616	0.641
Valeric acid	1.13	1.19	1.13	1.18	1.07	1.24	0.09	0.718	0.767	0.325	0.174	0.203	0.865	0.132
Total SCFA	40.85	44.34	41.73	43.46	41.77	43.41	2.43	0.486	0.728	0.742	***0.074***	0.649	0.738	0.289

^1^ Values are means; the SE of all groups is shown; *n* = 24/group (not enough colonic digesta of all piglets available for analyses). Colon digesta samples were obtained at 2 h after oral supplementation with Gln or Ala and milk replacer, and snap-frozen in liquid nitrogen. ^2^ ANOVA *F* test; significant differences (*p* < 0.05) are marked in bold, trends (*p* < 0.1) are marked in italic and bold. Ala = Alanine; BiW = birthweight; Gln = Glutamine; LBW = low birthweight; NBW = normal birthweight; SE = standard error; Supp = supplementation group.

**Table 4:** Microbial diversity indices and relative abundance of bacterial phyla in the colon digesta of male suckling piglets ^1^.

	Supp		BiW		Age			*p* Values ^2^						
Item, %	Gln	Ala	LBW	NBW	5 d	12 d	SEM	Supp	BiW	Age	Supp × BiW	Suppl × Age	BiW × Age	Suppl × BiW × Age
Richness ^3^	179	167	172	173	164	181	5.56	0.391	0.974	0.114	0.794	0.343	0.204	0.550
Shannon.Index ^3^	3.73	3.72	3.72	3.72	3.67	3.77	0.05	0.792	0.956	0.389	0.694	0.763	0.657	0.633
Evenness ^3^	0.72	0.73	0.72	0.73	0.72	0.73	0.01	0.613	0.801	0.621	0.867	0.775	0.959	0.695
Firmicutes	68.8	66.6	64.4	71.1	65.1	70.4	1.82	0.429	**0.049**	0.156	**0.009**	0.429	0.105	**0.032**
Bacteroidetes	22.1	21.9	23.6	20.4	23.8	20.2	1.40	0.301	0.423	***0.052***	0.412	0.175	0.180	0.420
Fusobacteria	3.75	6.42	5.71	4.35	6.89	3.17	1.17	0.538	0.374	0.362	0.371	0.670	0.625	0.635
Proteobacteria	3.07	3.77	4.01	2.80	3.65	3.16	0.57	0.322	0.258	0.307	0.504	0.510	0.486	0.804
Verrucomicrobia	0.78	0.00	0.81	0.00	0.00	0.81	0.40	0.973	0.360	**0.002**	0.741	**0.025**	**0.009**	***0.098***
WPS-2	0.42	0.10	0.06	0.47	0.00	0.53	0.17	0.179	0.138	0.173	0.233	0.281	0.256	0.473
Actinobacteria	0.33	0.24	0.32	0.26	0.36	0.21	0.06	0.524	0.904	0.869	0.779	0.796	0.717	0.836
Spirochaetes	0.29	0.32	0.43	0.17	0.05	0.55	0.11	0.380	0.917	**0.003**	0.784	**0.024**	**0.019**	0.121
Planctomycetes	0.27	0.17	0.26	0.18	0.07	0.38	0.06	***0.054***	0.248	0.113	0.166	***0.074***	0.293	0.309
Tenericutes	0.12	0.04	0.10	0.06	0.00	0.16	0.05	0.590	0.582	**0.002**	0.897	**0.022**	**0.023**	0.198
Epsilonbacteraeota	0.03	0.23	0.19	0.06	0.03	0.23	0.08	0.465	0.819	**<0.001**	0.896	**0.004**	**0.006**	***0.054***
Kiritimatiellaeota	0.02	0.11	0.06	0.07	0.00	0.12	0.03	0.396	0.973	**0.021**	0.757	**0.037**	0.127	0.242
Lentisphaerae	0.01	0.04	0.02	0.03	0.05	0.01	0.01	0.937	***0.080***	0.230	0.145	0.499	***0.083***	0.191

^1^ Values are means of relative abundance; the SE for all groups is shown; *n* = 22/group (not enough colonic digesta of all piglets available for analyses). Colon digesta samples were obtained at 2 h after oral supplementation of Gln or Ala and milk replacer, and snap-frozen in liquid nitrogen. ^2^ Kruskal–Wallis test, asymptotic significance; significant differences (*p* < 0.05) are marked in bold, trends (*p* < 0.1) are marked in italic and bold ^3^ Calculated on amplicon sequence variant (ASV) level. Ala = Alanine; BiW = birthweight; Gln = Glutamine; LBW = low birthweight; NBW = normal birthweight; SE = standard error of the mean; Supp = supplementation group.

## Data Availability

The data presented in this study are available on request from the corresponding author.

## Supplementary Materials

The following supporting information can be downloaded at: https://www.mdpi.com/article/10.3390/microorganisms10101899/s1. Figure S1: Hierarchical clustering of bacterial genera in colon of male suckling piglets; Table S1: Morphometric measurements of the colon mucosa of 5 and 12-day old suckling piglets splitted; Table S2: Immunohistomorphometric measurements in the colon of 5 and 12-day old male suckling piglets splitted; Table S3: Goblet cells in colon of 5 and 12-day old male suckling piglets splitted; Table S4: IgA positive cells in the colon lamina propria of 12-day old male suckling piglets; Table S5: Concentrations of biogenic amines in the colon digesta of 5 and 12-d suckling piglets splitted; Table S6: SCFA concentrations in the colon digesta of 5 and 12-d old suckling piglets splitted; Table S7: Relative abundance of bacterial order in colon digesta of male suckling piglets; Table S8: Relative abundance of bacterial genera in colon digesta of male suckling piglets; Tabel S9: Diversity of bacterial abundance in the colon digesta of 5 and 12-d old suckling piglets splitted; Table S10: Relative abundance of bacterial phyla in the colon digesta of male suckling piglets splitted; Table S11: Relative abundance of bacterial order in the colon digesta of male suckling piglets splitted; Table S12: Relative abundance of bacterial genera in the colon digesta of male suckling piglets splitted.
