# Supplementary material for: Effects of Oral Glutamine Supplementation, Birthweight and Age on Colonic Morphology and Microbiome Development in Male Suckling Piglets
Source: Microorganisms. 2022 Sep 25;10(10):1899. doi: 10.3390/microorganisms10101899 (PMC9612066; doi:10.3390/microorganisms10101899)
Supplement: Supplementary file 1 [file microorganisms-10-01899-s001.zip › microorganisms-1910548-supplementary.pdf]

Table S1. Morphometric measurements of the colon mucosa of 5 and 12 – day old suckling piglets splitted[1]

		Ala		Gln			*p* values[2]			
**Item**	**Age-group**	**LBW**	**NBW**	**LBW**	**NBW**	**SE**	**Supp**	**BiW**	**Supp x BiW**	**Age**
CD, μm	5	207^{ace}	220^{be}	192^{ade}	220^{be}	1.421	**0.005**	**0.000**	**0.003**	**0.000**
	12	262^{f}	254^{f}	276^{bf}	260^{af}	2.334	**0.037**	**0.008**	0.374	
CA, μm²	5	9492^{ce}	9811^{e}	8679^{de}	9573^{e}	88.15	**0.002**	**0.000**	***0.097***	**0.000**
	12	12267^{cf}	12401^{f}	13418^{bdf}	12129^{af}	168.1	0.189	***0.084***	**0.034**	

[1]Values are Means, the SE of all groups is shown; n = 12 / group (5, 12 d).
Colon samples were obtained at 2 h after oral administration of milk replacer and Gln or Ala supplement and fixed in Formalin.
[2]ANOVA *F* test; Significant differences ($p < 0.05$) are marked in bold, trends ($p < 0.1$) are marked in italic and bold
F-tests, none of the other fixed effects or their combination were significant ($p < 0.05$)
[a, b]Labeled Means in a row within one BiW group and one age group without a common letter differ, $p < 0.05$ (Tukey-test).
[c,d]Labeled Means in a row within one supplementation group and one age group without a common letter differ, $p < 0.05$ (Tukey-test).
[e,f]Labeled Means in a column within one supplementation group and BiW group without a common letter differ, $p < 0.05$ (Tukey-test).
Ala = Alanine; BiW = birthweight, CA= crypt area; CD = crypt depth; Gln = Glutamine; LBW = low birthweight; NBW = normal birthweight; SE = standard error; Supp = supplementation group

Table S2. Immunohistomorphometric measurements in the colon of 5 and 12-day old male suckling piglets splitted[1]

		Ala		Gln			*p* values[2]			
Item	Age-group	LBW	NBW	LBW	NBW	SE	Supp	BiW	Suppl x BiW	Age
	5	1.13^{e}	1.10^{e}	1.10^{e}	1.27^{e}	0.065	0.612	0.612	0.446	
CD3⁺ IEL										**0.043**
	12	2.03^{acf}	2.77^{bf}	2.82^{df}	2.84^{f}	0.094	**0.020**	**0.040**	***0.054***	
	5	5.13^{e}	5.93^{e}	6.03^{e}	6.48^{e}	0.166	**0.028**	***0.058***	0.595	
CD3⁺ lamina propria										0.603
	12	11.0^{f}	11.1^{f}	11.2^{f}	11.6^{f}	0.223	0.442	0.559	0.801	

[1]Values are Means of $CD3^{+}$ positive cells per 100 enterocytes and per 10 000 μm^2 lamina propria next to the crypts, the SE of all groups is shown; n = 12 / group (5, 12 d).
Colon samples were obtained at 2 h after oral administration of milk replacer and Gln or Ala supplement and fixed in Formalin.
[2]ANOVA *F* test; Significant differences ($p < 0.05$) are marked in bold, trends ($p < 0.1$) are marked in italic and bold
F-tests, none of the fixed effects or their combination were significant ($p < 0.05$)
[a, b]Labeled Means in a row within one BiW group and one age group without a common letter differ, $p < 0.05$ (Tukey-test).
[c,d]Labeled Means in a row within one supplementation group and one age group without a common letter differ, $p < 0.05$ (Tukey-test).
[e,f]Labeled Means in a column within one supplementation group and BiW group without a common letter differ, $p < 0.05$ (Tukey-test).
Ala = Alanine; BiW = birthweight; Gln = Glutamine; LBW = low birthweight; NBW = normal birthweight; SE = standard error; Supp = supplementation group

Table S3. Goblet cells in colon of 5 and 12-day old male suckling piglets splitted[1]

			Ala		Gln			*p* values[2]
**Item**		**Age-group**	**LBW**	**NBW**	**LBW**	**NBW**	**SE**	**Age**
AB-PAS Staining								
Crypt	Acid	5	23.2	18.3	21.6	23.7	1.453	0.145
		12	22.1	24.0	24.0	29.3	1.549	
	Neu	5	74.0	74.2	79.1	74.3	2.772	**0.006**
		12	71.1	49.1	59.4	63.8	4.266	
	Mixed	5	93.5	94.9[e]	102	95.7	2.593	**0.000**
		12	86.4	61.5[f]	75.2	76.0	3.952	
	Total	5	191	187	202	194	5.163	**0.001**
		12	179	134	159	169	8.552	

[1]Values are Means, the SE for all groups is shown; $n = 12$ / group (5, 12 d).
Colon samples were obtained at 2 h after oral administration of milk replacer and Gln or Ala supplement and fixed in Formalin.
[2]ANOVA *F* test; Significant differences ($p < 0.05$) are marked in bold, trends ($p < 0.1$) are marked in italic and bold
F-tests, none of the other fixed effects or their combination were significant ($p < 0.05$)
[e,f] Labeled Means in a column within one treatment group and BiW group without a common letter differ, $p < 0.05$ (Tukey-test).
Ala = Alanine; Acid = acidic mucins; Gln = Glutamine; LBW = low birth weight; NBW = normal body weight; Neu = neutral mucins. Mixed = mixed neutral and acidic mucins; SE = standard error; Supp = supplementation; Total = total number of AB-PAS positive goblet cells

Table S4. IgA positive cells in the colon lamina propria of 12-day old male suckling piglets[1]

Item	Age-group	Ala		Gln		SE
		LBW	NBW	LBW	NBW	
Lamina propria next to the crypt	12	8.25	9.17	7.83	8.5	0.541

[1]Values are Means of IgA positive cells per 10000 μm^2 of Lamina propria, the SE for all groups is shown; n = 12 / group (5, 12 d). Colon samples were obtained at 2 h after oral administration of milk replacer and Gln or Ala supplement and fixed in Formalin. ANOVA F test.

F-tests, none of the fixed effects or their combination were significant *(p < 0,05)*

Ala = Alanine; Gln = Glutamine; LBW = low birthweight; NBW = normal birthweight; SE = standard error

Table S5. Concentrations of biogenic amines in the colon digesta of 5 and 12-d suckling piglets splitted[1]

		**Ala**		**Gln**			***p* values[2]**			
**Item, µmol/g wet weight**	**Age**	**LBW**	**NBW**	**LBW**	**NBW**	**SE**	**Supp**	**BiW**	**Suppl x BiW**	**Age**
Propylamine	5	0.12	0.10	0.02	0.03	0.03	0.215	0.998	0.863	0.196
	12	0.02	0.04	0.03	0.04	0.01	0.578	0.124	0.581	
Putrescine	5	0.79	0.98	0.31	0.71	0.13	0.200	0.298	0.689	**0.020**
	12	0.41	0.35	0.40	0.45	0.04	0.617	0.942	0.550	
Histamine	5	0.03	0.15	0.14	0.05	0.04	0.989	0.881	0.383	0.508
	12	0.03	0.07	0.08	0.09	0.01	0.242	0.310	0.582	
Cadaverine	5	0.03	0.04	0.03	0.82	0.14	0.051	**0.048**	***0.053***	0.388
	12	0.08	0.05	0.02	0.28	0.07	0.529	0.416	0.314	
Spermidine	5	0.43	0.39	0.60	0.37	0.04	0.329	0.100	0.211	**0.002**
	12	0.17	0.24	0.33	0.33	0.03	0.020	0.393	0.432	
Spermine	5	0.03	0.02	0.03	0.03	0.00	0.621	0.870	0.608	0.773
	12	0.01	0.04	0.04	0.03	0.00	0.284	0.431	**0.038**	
Tyramine	5	0.12	0.10	0.06	0.64	0.11	0.261	0.200	0.174	***0.055***
	12	0.01	0.04	0.04	0.05	0.01	0.426	0.580	0.828	
Total biogenic amine	5	1.55	1.79	1.18	2.65	0.23	0.393	**0.029**	***0.076***	**0.002**
	12	0.73	0.83	0.94	1.28	0.12	0.191	0.374	0.622	

[1]Values are Means,the SE of all groups is shown; $n = 2$ / group (5 d) , $n = 3$ / group (12 d).
Colonic digesta samples were obtained at 2 h after oral administration of milk replacer and Gln or Ala supplement and snap frozen in liquid nitrogen.
[2]ANOVA *F* test; Significant differences ($p < 0.05$) are marked in bold, trends ($p < 0.1$) are marked in italic and bold
F-tests, none of the other fixed effects or their combination were significant ($p < 0.05$)
Ala = Alanine; BiW = birthweight; Gln = Glutamine; LBW = low birthweight; NBW = normal birthweight; SE = standard error; Supp = supplementation group

Table S6. SCFA concentrations in the colon digesta of 5 and 12-d old suckling piglets splitted[1]

		Ala		Gln			p values[2]
**Item, mmol/L**	**Age**	**LBW**	**NBW**	**LBW**	**NBW**	**SE**	**Suppl x BiW**
Acetic acid	5	27.46	26.5	25.13	29.45	2.12	0.570
	12	29.13	21.5	25.33	28.85	1.86	0.156
Propionic acid	5	7.69	7.48	7.64	9.49	0.84	0.571
	12	10.25	6.36	7.19	13.63	1.44	***0.084***
i-butyric acid	5	1.39	1.40	1.26	1.52	0.16	0.708
	12	1.52	1.25	1.37	1.79	0.11	0.148
n-butyric acid	5	2.58	3.04	2.73	3.10	0.26	0.939
	12	4.16	2.21	2.64	5.92	0.73	***0.084***
i-valeric acid	5	1.14	1.34	1.05	1.41	0.14	0.793
	12	1.34	1.17	1.37	1.71	0.10	0.209
n-valeric acid	5	1.10	1.20	0.96	1.02	0.12	0.920
	12	1.34	0.86	1.12	1.65	0.12	**0.040**
Total SCFA	5	41.37	41.0	38.78	46.0	3.48	0.617
	12	47.7	33.3	39.0	53.6	3.46	**0.039**

[1]Values are Means,the SE of all groups is shown; *n* = 5 / group (5, 12 d).
Colonic digesta samples were obtained at 2 h after oral administration of milk replacer and Gln or Ala supplement and snap frozen in liquid nitrogen.
[2]ANOVA *F* test; Significant differences ($p < 0.05$) are marked in bold, trends ($p < 0.1$) are marked in italic and bold
F-tests, none of the other fixed effects or their combination were significant ($p < 0.05$)
Ala = Alanine; BiW = birthweight; Gln = Glutamine; LBW = low birthweight; NBW = normal birthweight; SE = standard error; Supp = supplementation group

Table S7. Relative abundance of bacterial order in colon digesta of male suckling piglets[1]

	Supp		BiW		Age			*p* values[2]						
Item, (%)	Gln	Ala	LBW	NBW	5d	12d	SEM	Supp	BiW	Age	Supp x BiW	Suppl x Age	BiW x Age	Suppl x BiW x Age
Clostridiales	35.2	30.0	30.2	35.2	29.6	35.8	1.83	0.455	0.684	0.767	0.474	0.808	0.967	0.692
Lactobacillales	31.7	35.2	32.4	34.4	34.0	32.8	2.05	0.253	0.116	***0.063***	0.262	0.137	*0.100*	0.258
Bacteroidales	22.1	21.9	23.6	20.4	23.8	20.2	1.40	0.301	0.423	***0.052***	0.412	0.175	0.180	0.420
Fusobacteriales	3.75	6.42	5.71	4.35	6.89	3.17	1.17	0.553	0.386	0.350	0.381	0.667	0.624	0.642
Pasteurellales	2.09	2.74	2.92	1.87	2.38	2.41	0.56	0.253	0.277	0.733	0.419	0.518	0.676	0.770
Erysipelotrichales	0.90	0.44	0.80	0.55	0.83	0.52	0.13	0.150	0.531	0.660	0.473	0.351	0.657	0.324
Selenomonadales	0.86	0.79	0.90	0.75	0.53	1.12	0.21	0.281	0.166	0.435	0.231	0.467	0.310	0.409
Enterobacteriales	0.44	0.63	0.68	0.38	0.66	0.40	0.13	0.322	0.767	0.956	0.253	0.712	0.970	0.702
unknown WPS-2	0.42	0.10	0.06	0.47	0.00	0.53	0.17	0.973	0.360	**0.002**	0.741	**0.025**	**0.009**	***0.098***
Bradymonadales	0.34	0.19	0.19	0.34	0.46	0.07	0.13	0.300	0.549	0.609	**0.024**	0.300	0.877	***0.062***
Spirochaetales	0.29	0.32	0.43	0.17	0.05	0.55	0.11	0.122	0.903	***0.054***	0.355	**0.025**	0.291	0.193
Pirellulales	0.27	0.17	0.26	0.18	0.07	0.38	0.06	0.187	0.435	0.297	0.440	0.413	0.606	0.618
Coriobacteriales	0.22	0.15	0.20	0.17	0.30	0.07	0.05	0.380	0.917	**0.003**	0.784	**0.024**	**0.019**	0.121
unknown Firmicutes	0.15	0.16	0.14	0.16	0.11	0.20	0.02	***0.054***	0.248	0.113	0.166	***0.074***	0.293	0.309
Desulfovibrionales	0.12	0.15	0.16	0.11	0.10	0.17	0.02	0.590	0.582	**0.002**	0.897	**0.022**	**0.023**	0.198
Mollicutes_RF39	0.12	0.03	0.10	0.06	0.00	0.15	0.05	0.850	0.911	***0.058***	0.982	0.299	**0.039**	0.195
Betaproteobacteriales	0.08	0.04	0.05	0.06	0.04	0.08	0.02	0.947	0.396	**0.002**	0.707	**0.025**	**0.010**	***0.068***
Micrococcales	0.07	0.06	0.07	0.06	0.04	0.09	0.01	0.965	0.974	***0.092***	0.875	0.372	0.365	0.493
Campylobacterales	0.03	0.23	0.19	0.06	0.03	0.23	0.08	0.396	0.973	**0.021**	0.757	**0.037**	0.127	0.242
Corynebacteriales	0.02	0.03	0.02	0.03	0.00	0.05	0.01	0.622	0.300	**0.005**	0.691	**0.037**	**0.022**	0.178
WCHB1-41	0.02	0.11	0.06	0.07	0.00	0.12	0.03	0.834	0.402	0.880	***0.096***	0.889	0.863	0.408
Actinomycetales	0.02	0.01	0.02	0.01	0.02	0.01	0.00	0.701	***0.062***	0.468	0.120	0.876	0.139	0.292
Victivallales	0.01	0.03	0.02	0.02	0.03	0.01	0.01	0.482	0.635	**0.037**	0.574	***0.077***	0.127	0.246
Bacillales	0.00	0.01	0.01	0.01	0.00	0.01	0.00	0.172	0.341	0.155	0.355	0.259	0.376	0.542

Oligosphaerales	0.00	0.01	0.01	0.01	0.02	0.00	0.00	0.525	0.669	0.247	0.193	0.383	0.518	0.360

[1]Values are Means of relative abundance, the SE for all groups is shown; $n = 22$ / group.
Colon digesta samples were obtained at 2 h after oral administration of milk replacer and Gln or Ala supplementation and snap frozen in liquid nitrogen.
[2] Kruskal Wallis Test. asymptotic significance; Significant differences ($p < 0.05$) are marked in bold, trends ($p < 0.1$) are marked in italic and bold
Ala = Alanine; BiW = birthweight; Gln = Glutamine; LBW = low birthweight; NBW = normal birthweight; SE = standard error; Supp = supplementation group

Table S8. Relative abundance of bacterial genera in colon digesta of male suckling piglets[1]

	Supp		BiW		Age			*p* values[2]						
Item, (%)	Gln	Ala	LBW	NBW	5d	12d	SEM	Supp	BiW	Age	Supp x BiW	Suppl x Age	BiW x Age	Suppl x BiW x Age
Lactobacillus	30.7	34.0	31.1	33.5	32.5	32.1	2.00	0.482	0.546	0.991	0.505	0.835	0.937	0.704
Clostridium_sensu_stricto_1	9.12	9.12	8.36	9.88	9.65	8.59	0.88	0.524	0.637	0.546	0.700	0.753	0.711	0.857
unknown Muribaculaceae	5.27	3.03	4.71	3.69	6.82	1.58	0.85	0.982	0.835	**0.001**	0.854	**0.008**	**0.002**	**0.018**
Bacteroides	4.38	2.18	3.69	2.97	4.49	2.16	0.72	0.141	0.869	0.150	0.528	0.232	0.263	0.487
Fusobacterium	3.71	6.37	5.65	4.32	6.83	3.14	1.16	0.301	0.435	***0.052***	0.410	0.175	0.181	0.415
unknown Ruminococcaceae	3.41	2.94	2.53	3.84	2.44	3.93	0.43	0.429	0.177	0.223	0.379	0.546	0.142	0.224
Prevotella_2	3.14	3.94	3.60	3.44	3.44	3.61	0.94	1.000	0.636	0.917	0.963	0.926	0.117	0.958
Rikenellaceae_RC9_gut_group	3.05	4.13	3.60	3.54	3.00	4.14	0.75	0.248	0.660	0.462	0.664	0.508	0.790	0.781
Romboutsia	2.84	2.46	2.69	2.62	1.93	3.38	0.33	0.886	0.660	**0.010**	0.299	***0.082***	***0.056***	0.142
unknown Lachnospiraceae	2.70	2.15	1.68	3.19	3.79	1.08	0.55	0.660	0.287	**0.013**	0.562	**0.042**	**0.048**	0.157
Actinobacillus	1.95	2.62	2.73	1.82	2.22	2.32	0.54	0.262	0.258	0.800	0.389	0.495	0.675	0.737
Ruminococcaceae_UCG-002	1.69	1.69	1.55	1.83	0.88	2.51	0.29	0.708	0.545	**0.004**	0.827	**0.041**	**0.034**	0.246
Ruminococcaceae_UCG-005	1.41	0.99	1.11	1.31	0.19	2.23	0.27	0.391	0.758	**< 0.001**	0.679	**< 0.001**	**< 0.001**	**0.001**
Alloprevotella	1.38	2.09	1.92	1.52	0.70	2.74	0.46	0.252	0.904	**0.024**	0.443	***0.090***	0.161	0.297
Christensenellaceae_R-7_group	1.23	0.95	0.95	1.25	0.24	1.96	0.21	0.930	0.742	**< 0.001**	0.940	**< 0.001**	**< 0.001**	**0.002**

Lachnoclostridium	1.17	1.04	1.03	1.18	1.42	0.79	0.17	0.248	0.742	**0.022**	0.597	***0.083***	0.149	0.359
unknown Clostridiales_vadinBB60_group	1.08	0.69	1.44	0.35	0.22	1.57	0.29	0.293	0.288	**< 0.001**	0.440	**0.001**	**0.001**	**0.011**
Blautia	1.00	0.35	0.49	0.89	1.10	0.28	0.21	0.289	0.536	**0.022**	0.608	***0.093***	0.117	0.384
unknown F082	0.97	1.20	1.31	0.86	0.00	2.16	0.44	0.659	0.325	**< 0.001**	0.710	**0.003**	**0.010**	**0.029**
Ruminococcaceae_NK4A214_group	0.86	0.73	0.76	0.84	0.55	1.05	0.11	0.725	0.767	**0.018**	0.973	0.103	0.101	0.428
unknown p-2534-18B5_gut_group	0.85	0.48	0.53	0.82	0.03	1.32	0.26	0.703	0.341	**< 0.001**	0.575	**< 0.001**	**0.008**	**0.006**
Ruminococcus_2	0.84	0.35	0.66	0.55	0.69	0.52	0.13	0.167	0.648	**0.041**	0.545	***0.059***	0.216	0.335
Prevotellaceae_NK3B31_group	0.82	2.14	1.58	1.32	2.24	0.66	0.35	0.101	0.956	***0.091***	0.436	***0.063***	0.413	0.385
Streptococcus	0.78	0.95	1.11	0.62	1.28	0.44	0.17	0.843	0.435	**0.006**	0.430	***0.054***	**0.038**	0.133
Family_XIII_AD3011_group	0.70	0.65	0.64	0.71	0.37	0.97	0.11	0.965	0.684	**0.008**	0.982	***0.060***	**0.037**	0.219
Prevotellaceae_UCG-003	0.67	0.34	0.71	0.30	0.53	0.49	0.15	0.827	0.330	***0.053***	0.407	0.251	0.161	0.385
Parabacteroides	0.63	1.48	1.04	1.04	1.63	0.44	0.35	0.965	0.307	**< 0.001**	0.598	**0.010**	**0.004**	***0.054***
Roseburia	0.60	0.33	0.55	0.40	0.13	0.81	0.25	0.374	0.354	**0.009**	0.637	**0.045**	**0.042**	0.148
Dorea	0.53	0.33	0.33	0.54	0.28	0.58	0.07	0.242	***0.066***	**0.021**	0.146	**0.012**	**0.014**	**0.018**
Terrisporobacter	0.52	0.30	0.37	0.45	0.30	0.53	0.08	0.266	0.676	0.166	0.213	0.241	0.539	0.394
Ruminiclostridium_9	0.48	0.28	0.37	0.40	0.24	0.53	0.06	0.257	0.590	0.170	0.660	***0.074***	0.471	0.373
Phascolarctobacterium	0.48	0.41	0.45	0.45	0.17	0.73	0.13	***0.086***	0.191	**0.017**	***0.055***	**0.031**	***0.053***	**0.049**
Escherichia/Shigella	0.44	0.62	0.67	0.38	0.65	0.40	0.13	0.150	0.531	0.660	0.473	0.351	0.657	0.324
unknown WPS-2	0.42	0.10	0.06	0.47	0.00	0.53	0.17	0.973	0.360	**0.002**	0.741	**0.025**	0.566	***0.098***
Turicibacter	0.41	0.24	0.41	0.24	0.43	0.22	0.10	0.689	0.172	0.226	0.255	0.564	0.360	0.577
Marvinbryantia	0.40	0.09	0.10	0.41	0.13	0.38	0.11	0.153	0.533	0.416	0.222	0.433	0.712	0.556
Intestinimonas	0.39	0.34	0.32	0.41	0.32	0.41	0.05	0.194	0.496	0.170	0.500	0.306	0.335	0.594
Ruminococcaceae_UCG-004	0.34	0.24	0.32	0.26	0.17	0.41	0.05	0.552	0.886	***0.057***	0.946	0.255	0.304	0.706
Subdoligranulum	0.34	0.10	0.11	0.33	0.09	0.35	0.13	0.605	0.834	0.547	0.950	0.887	0.216	0.990
unknown Bradymonadales	0.34	0.19	0.19	0.34	0.46	0.07	0.13	0.300	0.549	0.609	**0.024**	0.300	**0.008**	***0.062***
Oscillospira	0.32	0.29	0.35	0.27	0.15	0.47	0.06	0.725	0.509	**0.003**	0.830	**0.029**	**0.016**	0.120

Tyzzerella	0.31	0.31	0.26	0.36	0.45	0.18	0.06	0.851	0.280	**0.012**	0.485	**0.031**	**0.046**	**0.020**
Peptostreptococcus	0.31	0.18	0.37	0.12	0.46	0.04	0.11	0.123	*0.087*	**0.002**	0.143	**0.008**	**0.004**	**0.026**
Clostridium_sensu_stricto_2	0.28	0.86	0.74	0.38	0.95	0.17	0.22	0.843	0.338	**0.009**	0.799	**0.047**	**0.042**	0.230
p-1088-a5_gut_group	0.27	0.17	0.26	0.18	0.07	0.38	0.06	0.590	0.582	**0.002**	0.897	**0.022**	0.902	0.198
Holdemanella	0.25	0.05	0.19	0.13	0.20	0.12	0.06	0.699	0.662	**0.033**	0.849	0.166	0.525	0.569
Veillonella	0.24	0.18	0.31	0.11	0.36	0.06	0.06	0.176	0.106	**0.003**	0.215	**0.014**	**0.008**	**0.039**
Butyricimonas	0.20	0.26	0.28	0.17	0.23	0.23	0.08	0.336	0.508	0.284	0.701	0.295	0.607	0.678
Ruminococcaceae_UCG-010	0.17	0.16	0.14	0.19	0.17	0.17	0.02	0.775	0.545	0.750	0.785	0.969	0.902	0.939
Eisenbergiella	0.17	0.17	0.19	0.15	0.30	0.04	0.05	0.584	0.657	**< 0.001**	0.891	**< 0.001**	**< 0.001**	**0.001**
Sphaerochaeta	0.17	0.24	0.33	0.08	0.05	0.35	0.09	0.392	0.991	**0.015**	0.746	***0.081***	0.607	0.230
unknown Clostridiaceae_1	0.16	0.28	0.19	0.24	0.29	0.14	0.04	*0.091*	0.501	***0.085***	0.342	***0.089***	0.242	0.233
unknown Lactobacillales	0.16	0.18	0.12	0.21	0.11	0.22	0.04	0.302	0.247	0.252	0.266	0.488	0.412	0.587
Collinsella	0.15	0.10	0.14	0.12	0.23	0.02	0.05	0.576	0.694	**0.032**	0.897	0.109	**< 0.001**	0.274
Prevotella	0.15	0.06	0.06	0.16	0.15	0.06	0.06	0.882	0.968	0.608	0.867	0.962	0.306	0.912
unknown Firmicutes	0.15	0.16	0.14	0.16	0.11	0.20	0.02	0.965	0.974	***0.092***	0.875	0.372	0.365	0.493
Epulopiscium	0.14	0.13	0.13	0.15	0.18	0.10	0.03	0.302	0.799	0.542	0.269	0.566	0.911	0.629
unknown Erysipelotrichaceae	0.13	0.08	0.08	0.13	0.13	0.08	0.04	0.911	0.527	0.973	0.183	0.854	***0.090***	0.117
unknown Pasteurellaceae	0.13	0.10	0.16	0.06	0.16	0.07	0.04	0.286	0.973	0.353	0.765	0.572	0.304	0.936
Treponema_2	0.12	0.08	0.10	0.09	0.00	0.20	0.04	0.291	0.644	**< 0.001**	0.689	**0.002**	0.454	**0.038**
unknown Mollicutes_RF39	0.12	0.03	0.10	0.06	0.00	0.15	0.05	0.947	0.396	**0.002**	0.707	**0.025**	**0.014**	***0.068***
unknown Prevotellaceae	0.11	0.09	0.07	0.13	0.09	0.11	0.03	0.694	***0.063***	0.510	**0.049**	0.332	**0.046**	0.106
Desulfovibrio	0.11	0.13	0.13	0.11	0.10	0.14	0.02	0.153	0.503	0.489	0.432	0.454	0.805	0.707
Lachnospiraceae_UCG-004	0.10	0.07	0.09	0.09	0.04	0.14	0.02	0.548	0.686	**< 0.001**	0.897	**0.003**	**0.014**	**0.031**
Hungatella	0.10	0.10	0.10	0.10	0.15	0.06	0.04	0.764	0.690	**0.003**	0.941	**0.008**	**0.019**	***0.083***
unknown Clostridiales	0.09	0.08	0.06	0.10	0.05	0.12	0.01	0.947	0.134	**0.011**	0.217	***0.074***	**0.019**	***0.066***
Clostridium_sensu_stricto_4	0.09	0.07	0.08	0.08	0.03	0.12	0.02	0.573	0.445	**0.010**	0.219	**0.040**	0.413	***0.068***

Prevotella_9	0.08	0.09	0.07	0.11	0.13	0.05	0.03	0.196	0.918	0.605	0.641	0.573	0.753	0.958
Oscillibacter	0.08	0.11	0.06	0.13	0.10	0.09	0.03	0.434	0.434	0.308	0.567	0.317	0.525	0.169
GCA-900066225	0.08	0.06	0.08	0.06	0.12	0.02	0.01	0.307	0.973	**< 0.001**	0.744	**< 0.001**	**< 0.001**	**0.003**
Coprococcus_3	0.08	0.05	0.06	0.07	0.04	0.09	0.01	0.459	0.362	**0.029**	0.240	0.149	0.365	0.150
Erysipelotrichaceae_UCG-004	0.07	0.04	0.08	0.03	0.03	0.08	0.03	0.411	0.185	0.377	0.209	0.630	**0.041**	0.574
UBA1819	0.07	0.06	0.08	0.06	0.09	0.04	0.02	0.891	0.322	**0.002**	0.685	**0.021**	**0.011**	***0.072***
Rothia	0.07	0.06	0.07	0.06	0.04	0.09	0.01	0.850	0.911	***0.058***	0.982	0.299	**0.039**	0.195
Alistipes	0.06	0.07	0.12	0.01	0.08	0.06	0.03	0.368	**0.043**	0.680	**0.030**	0.764	**0.004**	0.205
Intestinibacter	0.06	0.05	0.06	0.06	0.03	0.09	0.02	0.264	0.501	**0.034**	0.296	0.115	0.412	0.321
Prevotellaceae_UCG-004	0.05	0.15	0.08	0.11	0.08	0.12	0.04	1.000	0.289	0.166	0.471	0.365	**0.001**	0.393
Candidatus_Soleaferrea	0.05	0.04	0.04	0.05	0.01	0.08	0.01	0.466	0.653	**< 0.001**	0.550	**0.007**	**0.019**	**0.034**
unknown Family_XIII	0.05	0.05	0.05	0.05	0.09	0.01	0.02	0.389	0.734	**< 0.001**	0.835	**0.006**	***0.090***	***0.082***
unknown Carnobacteriaceae	0.05	0.10	0.06	0.09	0.14	0.01	0.02	***0.055***	0.200	**0.004**	0.110	**0.006**	0.232	**0.018**
Prevotellaceae_UCG-001	0.05	0.00	0.01	0.05	0.00	0.06	0.02	0.172	0.894	**0.002**	0.267	**0.004**	0.372	**0.013**
dgA-11_gut_group	0.05	0.04	0.05	0.05	0.03	0.06	0.01	0.636	0.725	0.489	0.947	0.771	0.360	0.375
GCA-900066575	0.05	0.01	0.04	0.01	0.01	0.05	0.02	0.200	1.000	***0.051***	0.340	***0.096***	0.508	0.236
Sutterella	0.05	0.04	0.05	0.03	0.03	0.05	0.01	0.430	0.619	0.865	***0.052***	0.815	0.911	0.258
Lachnospiraceae_UCG-010	0.04	0.05	0.04	0.06	0.03	0.07	0.01	0.509	0.585	***0.092***	0.862	0.270	0.994	0.638
Hydrogenoanaerobacterium	0.04	0.04	0.05	0.04	0.07	0.02	0.01	0.973	0.928	**0.001**	0.970	**0.013**	**0.008**	**0.037**
Mogibacterium	0.04	0.05	0.05	0.04	0.01	0.08	0.01	0.629	0.603	**0.003**	0.829	**0.025**	**< 0.001**	***0.096***
Denitrobacterium	0.04	0.03	0.04	0.03	0.03	0.04	0.01	0.579	0.954	0.184	0.910	0.222	**0.042**	0.679
Butyricicoccus	0.04	0.04	0.04	0.04	0.03	0.04	0.01	0.353	0.882	0.982	0.372	0.831	0.994	0.862
unknown Paludibacteraceae	0.04	0.02	0.03	0.03	0.01	0.05	0.01	0.751	0.318	0.210	0.534	0.626	**0.031**	0.639
Flavonifractor	0.04	0.03	0.03	0.04	0.07	0.00	0.01	0.703	0.751	**< 0.001**	0.435	**< 0.001**	0.242	**< 0.001**
Negativibacillus	0.03	0.03	0.01	0.05	0.06	0.00	0.01	0.860	**0.020**	**0.002**	0.138	**0.023**	0.351	**0.019**
unknown Fusobacteriaceae	0.03	0.05	0.05	0.03	0.06	0.02	0.01	0.257	0.597	**0.009**	0.635	**0.036**	0.712	0.242
Faecalibacterium	0.03	0.01	0.01	0.04	0.03	0.02	0.01	0.522	**0.039**	0.887	0.197	0.777	***0.082***	0.439

H1	0.03	0.02	0.04	0.01	0.00	0.05	0.01	0.654	0.611	**0.019**	0.873	0.118	**< 0.001**	0.490
Sarcina	0.03	0.02	0.01	0.05	0.05	0.00	0.01	0.725	0.187	0.165	0.581	0.533	***0.059***	0.495
Coprococcus_1	0.03	0.01	0.01	0.03	0.00	0.03	0.01	0.452	0.431	***0.081***	0.637	***0.099***	**0.021**	0.197
Howardella	0.03	0.05	0.05	0.03	0.07	0.02	0.01	0.129	0.777	**0.002**	0.157	**0.009**	**0.019**	**0.011**
Eubacterium	0.03	0.01	0.03	0.01	0.02	0.02	0.01	0.279	0.932	0.560	0.434	0.582	**0.031**	0.734
Lachnospiraceae_NC2004_group	0.03	0.05	0.04	0.03	0.02	0.05	0.01	0.403	0.925	0.173	0.700	0.461	***0.060***	0.411
unknown Bacteroidales	0.03	0.03	0.02	0.04	0.04	0.02	0.01	0.918	0.289	0.155	0.769	0.563	0.175	0.679
Ruminococcaceae_UCG-009	0.02	0.02	0.02	0.02	0.02	0.02	0.01	0.233	0.657	***0.097***	0.296	0.227	**0.011**	0.440
Campylobacter	0.02	0.20	0.18	0.03	0.00	0.22	0.08	0.772	0.176	**0.022**	0.484	0.150	0.495	0.384
Lachnospiraceae_FCS020_group	0.02	0.01	0.01	0.02	0.01	0.02	0.01	0.310	0.349	0.557	0.574	0.360	**0.047**	0.626
Corynebacterium_1	0.02	0.03	0.02	0.03	0.00	0.05	0.01	0.622	0.300	**0.005**	0.691	**0.037**	0.433	0.178
Ruminococcaceae_UCG-013	0.02	0.02	0.02	0.02	0.01	0.03	0.00	0.568	0.865	***0.054***	0.939	0.198	**0.008**	0.595
unknown Peptostreptococcaceae	0.02	0.03	0.02	0.03	0.01	0.04	0.01	0.756	0.359	**0.007**	0.810	***0.057***	0.546	0.222
unknown WCHB1-41	0.02	0.11	0.06	0.07	0.00	0.12	0.03	0.396	0.973	**0.021**	0.757	**0.037**	***0.063***	0.242
Actinomyces	0.02	0.01	0.02	0.01	0.02	0.01	0.00	0.252	0.472	***0.091***	0.565	0.208	**< 0.001**	0.519
Ruminococcus_1	0.02	0.01	0.02	0.01	0.00	0.03	0.00	0.468	0.451	**< 0.001**	0.778	**< 0.001**	***0.074***	**0.016**
Pseudoflavonifractor	0.02	0.02	0.03	0.01	0.01	0.03	0.01	0.812	0.823	0.762	0.516	0.433	0.835	0.599
Peptococcus	0.02	0.00	0.01	0.01	0.00	0.02	0.00	***0.081***	0.318	0.962	0.237	0.181	***0.090***	0.444
Ruminococcaceae_UCG-014	0.02	0.02	0.01	0.03	0.00	0.04	0.01	0.560	0.415	**0.016**	0.799	***0.052***	***0.093***	0.256
Enterococcus	0.01	0.00	0.00	0.01	0.00	0.01	0.00	0.232	0.738	0.792	0.397	0.653	0.564	0.815
Family_XIII_UCG-001	0.01	0.01	0.01	0.01	0.01	0.01	0.00	0.153	0.424	0.777	0.302	0.546	**0.039**	0.683
Mannheimia	0.01	0.02	0.03	0.00	0.01	0.03	0.01	0.499	***0.075***	0.925	0.302	0.334	**< 0.001**	0.358
Fournierella	0.01	0.01	0.01	0.01	0.02	0.00	0.00	0.611	0.763	**0.001**	0.842	**0.010**	0.969	0.105
Ruminiclostridium_5	0.01	0.00	0.01	0.01	0.02	0.00	0.00	0.428	0.988	**< 0.001**	0.887	**0.004**	***0.054***	***0.058***
Ruminococcaceae_UCG-003	0.01	0.00	0.01	0.00	0.01	0.00	0.00	0.824	0.536	0.623	***0.072***	0.937	0.241	0.366
unknown Peptococcaceae	0.01	0.07	0.01	0.06	0.07	0.01	0.03	0.173	0.627	***0.080***	0.536	0.176	0.103	0.599
Clostridium_sensu_stricto_13	0.01	0.02	0.01	0.02	0.02	0.01	0.00	0.146	0.370	0.887	0.351	0.545	***0.083***	0.776

Asteroleplasma	0.01	0.00	0.01	0.00	0.00	0.01	0.00	0.699	0.635	0.765	0.885	0.922	0.854	0.989
Helicobacter	0.01	0.03	0.01	0.03	0.03	0.01	0.01	0.204	0.887	0.763	0.612	0.164	0.255	0.588
unknown vadinBE97	0.01	0.01	0.01	0.01	0.01	0.00	0.00	0.906	0.123	0.297	0.261	0.777	**< 0.001**	0.429
Anaerotruncus	0.01	0.00	0.00	0.01	0.00	0.01	0.00	0.985	0.409	0.431	0.324	0.891	**0.009**	0.630
Bilophila	0.01	0.02	0.02	0.00	0.00	0.02	0.01	0.360	0.103	**0.002**	0.250	**0.012**	**0.012**	**0.025**
unknown Atopobiaceae	0.01	0.00	0.00	0.00	0.01	0.00	0.00	0.751	0.646	0.623	0.934	0.911	**0.029**	0.994
unknown Desulfovibrionaceae	0.00	0.00	0.01	0.00	0.00	0.01	0.00	0.430	***0.095***	***0.069***	0.331	0.268	**0.008**	0.356
Victivallis	0.00	0.02	0.01	0.02	0.02	0.01	0.01	0.836	0.409	0.368	0.334	0.836	***0.089***	0.357
Pygmaiobacter	0.00	0.00	0.00	0.01	0.01	0.00	0.00	0.925	0.925	***0.088***	0.902	0.283	0.317	0.312
horsej-a03	0.00	0.01	0.01	0.01	0.02	0.00	0.00	0.482	0.635	**0.037**	0.574	***0.077***	0.299	0.246
Acetitomaculum	0.00	0.00	0.00	0.00	0.00	0.00	0.00	0.860	0.212	0.586	0.644	0.934	***0.074***	0.874
Caproiciproducens	0.00	0.00	0.00	0.00	0.00	0.00	0.00	0.745	0.730	0.149	0.934	0.287	0.831	0.539
Defluviitaleaceae_UCG-011	0.00	0.02	0.01	0.00	0.01	0.01	0.00	0.251	0.868	0.135	***0.099***	0.228	0.295	0.248
Staphylococcus	0.00	0.01	0.01	0.00	0.00	0.00	0.00	0.276	0.329	0.955	**0.028**	0.262	**0.001**	***0.058***
unknown Streptococcaceae	0.00	0.02	0.01	0.00	0.01	0.00	0.00	***0.053***	0.348	***0.095***	***0.053***	***0.058***	**0.013**	***0.068***
CAG-873	0.00	0.01	0.01	0.00	0.01	0.00	0.00	0.113	0.138	**0.019**	**0.015**	**0.015**	0.488	**< 0.001**

[1]Values are Means of relative abundance, the SE for all groups is shown; n = 22 / group.
Colon digesta samples were obtained at 2 h after oral administration of milk replacer and Gln or Ala supplementation and snap frozen in liquid nitrogen.
[2] Kruskal Wallis Test. asymptotic significance; Significant differences ($p < 0.05$) are marked in bold, trends ($p < 0.1$) are marked in italic and bold
Ala = Alanine; BiW = birthweight; Gln = Glutamine; LBW = low birthweight; NBW = normal birthweight; SEM = standard error of the Mean; Supp = supplementation group

Tabel S9. Diversity of bacterial abundance in the colon digesta of 5 and 12-d old suckling piglets splitted[1]

		**Ala**		**Gln**		
**Item**	**Age**	**LBW**	**NBW**	**LBW**	**NBW**	**SE**
Richness	5	154	166	156	178	7.622
	12	183	163	193	186	7.869
Shannon.Index	5	3.73	3.58	3.50	3.84	0.070
	12	3.81	3.769	3.82	3.69	0.080
Evenness	5	0.74	0.70	0.69	0.74	0.108
	12	0.74	0.74	0.73	0.71	0.012

[1]Values are Means,the SE of all groups is shown ; $n = 5$ / group (5 and 12 d).
Kruskal – Wallis Test, none of the other fixed effects or their combination were significant *($p < 0.05$)*
Ala = Alanine; Gln = Glutamine; LBW = low birthweight; NBW = normal birthweight; SE = standard error

Table S10. Relative abundance of bacterial phyla in the colon digesta of male suckling piglets splitted[1]

		**Ala**		**Gln**			***p* values[2]**			
**Item, %**	**Age**	**LBW**	**NBW**	**LBW**	**NBW**	**SE**	**Supp**	**BiW**	**Suppl x BiW**	**Age**
Firmicutes	5	55.9^{a}	74.4^{bd}	67.0	63.5^{c}	2.52	0.854	0.110	**0.027**	0.156
Firmicutes	12	67.0	78.0	68.3	68.0	2.57	0.356	***0.085***	0.215	
Bacteroidetes	5	30.6	19.3	20.2	24.4	2.21	1.000	0.580	0.332	0.362
Bacteroidetes	12	21.2	17.1	21.8	20.7	1.69	0.424	0.356	0.645	
Fusobacteria	5	7.50	3.12	9.20	8.31	1.85	0.498	0.667	0.766	***0.052***
Fusobacteria	12	3.64	0.74	3.07	5.63	1.36	0.356	0.176	0.432	
Proteobacteria	5	5.29	2.76	3.27	3.23	0.78	0.902	0.424	0.877	0.307
Proteobacteria	12	2.78	1.46	4.59	3.94	0.84	0.268	0.460	0.632	
Actinobacteria	5	0.54	0.29	0.25	0.36	0.10	1.000	0.389	0.615	0.869
Actinobacteria	12	0.29	0.18	0.21	0.17	0.03	0.268	0.389	0.544	

Spirochates	5	0.09	0.10	0.06[e]	0.27	0.04	0.563	0.347	0.495	**0.003**
Spirochates	12	1.19	0.44	0.98[f]	0.32	0.27	0.619	0.368	0.795	
Epsilonbacteraeota	5	0.01	0.00	0.04	0.24	0.07	**0.031**	0.381	0.129	0.113
Epsilonbacteraeota	12	0.16	0.95	0.10	0.37	0.332	0.445	0.406	0.745	
Plantomycetes	5	0.23	0.41	0.19	0.11	0.055	0.563	0.772	0.899	**0.002**
Plantomycetes	12	0.78	0.63	0.35	0.25	0.141	0.951	0.757	0.985	
Lentisphaerae	5	0.06	0.07	0.05	0.23	0.051	0.485	0.834	0.566	0.230
Lentisphaerae	12	0.03[a]	0.00[b]	0.08	0.00	0.020	0.426	**0.009**	**0.036**	
WPS-2	5	0.00	0.00[-]	0.00	0.00	0.000	1.000	1.000	1.000	**0.002**
WPS-2	12	1.16	2.96[+]	0.12	0.96	0.887	0.942	0.193	0.594	
Verrucomicrobia	5	0.10	0.00	0.00	0.00	0.00	0.338	0.296	0.418	0.173
Verrucomicrobia	12	9.27	0.02	0.03	0.00	4.62	0.306	0.264	0.486	
Tenericutes	5	0.00	0.00	0.00	0.00[e]	0.00	1.000	1.000	1.000	**0.000**
Tenericutes	12	1.10	0.30	0.04[+]	0.22[f]	0.208	0.497	0.497	0.755	
Kritimatiellae	5	0.00	0.08	0.00	0.00	0.00	0.338	0.338	0.418	**0.021**
Kritimatiellae	12	0.22	0.17	0.37[+]	0.63	0.193	0.174	0.821	0.597	

[1]Values are Means of relative abundance, the SE for all groups is shown; *n* = 5 / group (5, 12 d).

Colon digesta samples were obtained at 2 h after oral administration of milk replacer and Gln or Ala supplement and snap frozen in liquid nitrogen.

[2] Kruskal Wallis Test, asymptotic significance (significant differences are marked in bold, trends in bold and italics)

Asymptotic significance, none of the other fixed effects or their combination were significant *($p < 0.05$)*

[a, b]Labeled Means in a row within one BiW group and one age group without a common letter differ, $P < 0.05$ (Mann-Whitney-U-test).

[c,d]Labeled Means in a row within one supplementation group and one age group without a common letter differ, $p < 0.05$ (Mann-Whitney-U-test).

[e,f]Labeled Means in a column within one supplementation group and BiW group without a common letter differ, $p < 0.05$ (Mann-Whitney-U-test).

[+,-] Labeled Means in a column within one supplementation group and BiW group without a common letter differ, $p < 0.1$ (Mann-Whitney-U-test).

Ala = Alanine; BiW = birthweight; Gln = Glutamine; LBW = low-birthweight; NBW = normal birthweight; SE = standard error; Supp = supplementation group

Table S11. Relative abundance of bacterial order in the colon digesta of male suckling piglets splitted[1]

		Ala		Gln			*p values*[2]			
**Item, %**	**Age**	**LBW**	**NBW**	**LBW**	**NBW**	**SE**	**Supp**	**BiW**	**Suppl X BiW**	**Age**
Lactobacillales	5	26.5	38.8	41.2	30.9	3.05	0.902	0.712	0.262	0.767
Lactobacillales	12	29.4	32.1	33.9	36.2	2.81	0.460	0.854	0.893	
Clostridiales	5	27.0	34.4	24.9	31.4	2.23	0.902	0.850	0.383	***0.063***
Clostridiales	12	35.2	44.2	32.8	29.9	2.81	0.157	0.460	0.368	
Bacteroidales	5	30.6	19.3	20.2	24.4	2.20	1.000	0.580	0.332	0.113
Bacteroidales	12	21.2	21.8	17.1	20.7	1.69	0.424	0.356	0.645	
Fusobacteriales	5	7.50	3.12	9.20	8.13	1.85	0.498	0.667	0.766	***0.052***
Fusobacteriales	12	3.64	0.74	3.07	5.63	1.36	0.356	0.176	0.432	
Pasteurellales	5	4.51	1.34	1.38	2.14	3.71	0.806	0.295	0.700	0.733
Pasteurellales	12	2.10	0.92	4.56	3.53	4.20	0.139	0.579	0.480	
Erysipelotrichales	5	1.76	0.80	0.32	0.79	0.24	0.805	0.782	0.698	0.435
Erysipelotrichales	12	0.91	0.55	0.42	0.14	0.14	0.157	***0.056***	0.111	
Selenomodales	5	0.89	0.39	0.39	0.42	0.11	0.712	0.538	0.448	0.956
Selenomodales	12	1.31	0.83	0.90	1.50	0.40	0.268	1.000	0.503	
Enterobacteriales	5	0.74	0.23	1.01	1.08	0.25	0.156	0.951	0.375	0.660
Enterobacteriales	12	0.83	0.24	0.46^{b}	0.15^{a}	0.14	0.667	*0.097*	0.154	
Coriobacteriales	5	0.53	0.28	0.28	0.34^{+}	0.12	0.902	0.902	0.955	***0.054***
Coriobacteriales	12	0.11^{c}	0.11	0.07^{d}	0.05^{-}	0.01	**0.004**	0.951	**0.035**	
Pirellulales	5	0.23	0.41	0.19^{-}	0.11	0.05	0.563	0.772	0.899	**0.002**
Pirellulales	12	0.78	0.63	0.35^{+}	0.23	0.14	0.951	0.757	0.985	
Unknown Firmicutes	5	0.12^{F}	0.15^{-}	0.22	0.09^{F}	0.02	0.665	0.665	0.435	*0.092*
Unknown Firmicutes	12	0.21^{E}	0.26^{+}	0.18	0.31^{E}	0.03	0.758	0.622	0.901	
Desulfovibronales	5	0.12	0.08	0.08	0.16	0.02	0.295	0.902	0.408	0.297
Desulfovibronales	12	0.16	0.14	0.28	0.08	0.04	0.460	0.424	0.683	

Actinomycetales	5	0.10	0.04	0.03	0.02	0.01	0.219	0.563	0.480	0.115
Actinomycetales	12	0.04	0.02	0.05	0.00	0.01	0.352	0.306	0.571	
Betaproteobacteriales	5	0.10	0.04	0.04	0.07	0.02	0.653	0.653	0.597	0.880
Betaproteobacteriales	12	0.20	0.33	0.05	0.08	0.06	0.507	0.528	0.199	
Spirochaetales	5	0.09	0.06	0.10^{E}	0.27	0.04	0.563	0.347	0.495	**0.003**
Spirochaetales	12	1.19	0.44	0.98^{F}	0.32	0.27	0.619	0.368	0.795	
Victivallales	5	0.05	0.01	0.01	0.26	0.06	0.880	0.623	0.455	0.468
Victivallales	12	$0.03^{\#}$	0.00^{*}	0.08	0.00	0.02	0.701	**0.019**	0.114	
Micrococcales	5	0.05	0.07	0.04^{E}	0.11	0.01	0.703	0.163	0.475	***0.058***
Micrococcales	12	0.20	0.07	$0.10^{F\#}$	0.09^{*}	0.02	1.000	***0.095***	0.362	
Betaproteobacteriales	5	0.10	0.04	0.04	0.07	0.02	0.653	0.653	0.597	0.880
Betaproteobacteriales	12	0.20	0.33	0.05	0.08	0.06	0.507	0.528	0.199	
Oligospherales	5	0.01	0.06	0.10	0.06^{+}	0.01	0.202	0.426	0.466	**0.037**
Oligospherales	12	0.03	0.00	0.00	0.00^{-}	0.00	0.338	0.338	0.418	
Campylobacterales	5	0.01^{z}	0.00	0.04^{x}	0.24	0.07	**0.031**	0.381	0.129	0.113
Campylobacterales	12	0.16	0.10	0.95	0.37	0.33	0.445	0.406	0.745	
Bradymondales	5	0.00^{*}	$2.23^{+\#}$	1.30^{+*}	$0.05^{+\#}$	0.67	0.743	0.763	*0.084*	0.609
Bradymondales	12	0.08^{b}	0.23^{a-}	$0.11^{-\#}$	0.03^{-*}	0.06	***0.054***	0.572	0.103	
Mollicutes_RF39	5	0.00	0.00	0.00	0.00^{F}	0.00	1.000	1.000	1.000	**0.002**
Mollicutes_RF39	12	1.09	0.30	0.03	0.22^{E}	0.25	1.000	0.247	0.477	
Unknown WPS-2	5	0.00	0.00^{-}	0.00	0.00	0.00	1.000	1.000	1.000	**0.002**
Unknown WPS-2	12	1.16	2.96^{+}	0.12	0.95	0.89	0.942	0.193	0.594	
WCHB1-41	5	0.00	0.08	0.00^{-}	0.00	0.00	0.338	0.338	0.418	**0.021**
WCHB1-41	12	0.22	0.17	0.37^{+}	0.63	0.19	0.174	0.821	0.597	
Corynebacteriales	5	0.00	0.00	0.00^{-}	0.00	0.00	1.000	1.000	1.000	**0.005**
Corynebacteriales	12	0.09	0.35	0.06^{+}	0.37	0.06	0.623	0.428	0.834	

[1]Values are Means of relative abundance, the largest SE is shown; *n* = 5 / group (5, 12 d).
Colon digesta samples were obtained at 2 h after oral administration of milk replacer and Gln or Ala supplement and snap frozen in liquid nitrogen.
[2] Kruskal Wallis Test, asymptotic significance (significant differences are marked in bold, trends in bold and italics)
Asymptotic significance, none of the other fixed effects or their combination were significant *($p < 0.05$)*
[a, b]Labeled Means in a row within one BiW group and one age group without a common letter differ, $p < 0.05$ (Mann-Whitney-U-test).

[c,d]Labeled Means in a row within one supplementation group and one age group without a common letter differ, $p < 0.05$ (Mann-Whitney-U-test).

[e,f]Labeled Means in a column within one supplementation group and BiW group without a common letter differ, $p < 0.05$ (Mann-Whitney-U-test).

[x,z] Labeled Means in a row within one BiW group and one age group without a different superscripts differ , $p < 0.1$ (Mann-Whitney-U-test).

[*,#]Labeled Means in a row within one supplementation group and one age group without a common letter differ, $p < 0.05$ (Mann-Whitney-U-test).

[+,-] Labeled Means in a column within one supplementation group and BiW group without a common letter differ, $p < 0.1$ (Mann-Whitney-U-test).

Ala = Alanine; BiW = birthweight; Gln = Glutamine; LBW = low birthweight; NBW = normal birthweight; SE = standard error; Supp = supplementation group

Table S12. Relative abundance of bacterial genera in the colon digesta of male suckling piglets splitted[1]

		Ala		Gln			*p* values[2]			
**Item, %**	**Age**	**LBW**	**NBW**	**LBW**	**NBW**	**SE**	**Suppl**	**BiW**	**BiwxSupp**	**Age**
Lactobacillus	5	24.9	37.6	38.7	29.6	0.032	1.000	0.538	0.245	0.991
Lactobacillus	12	28.8	31.4	33.0	35.7	2.761	0.424	0.758	0.859	0.991
unknown Muribaculaceae	5	11.1^{E}	7.33	6.13^{E}	2.61	0.052	0.176	0.140	0.238	**0.001**
unknown Muribaculaceae	12	0.58^{F}	2.09	1.28^{F}	2.53	0.368	0.356	*0.056*	0.118	**0.001**
Clostridium_sensu_stricto_1	5	7.82	10.7	8.91	11.0	0.078	0.389	0.268	0.569	0.546
Clostridium_sensu_stricto_1	12	7.55	10.3	9.23	6.94	1.284	1.000	0.622	0.738	0.546
Fusobacterium	5	7.43	3.06	9.10	8.09	0.051	0.498	0.712	0.757	***0.052***
Fusobacterium	12	3.62	0.74	3.03	5.59	1.351	0.356	0.176	0.432	***0.052***
Bacteroides	5	6.66	5.39	2.45	3.13	0.013	0.291	0.398	0.345	***0.061***
Bacteroides	12	3.29	2.17	2.15	0.82	0.620	0.356	0.429	0.624	***0.061***
Prevotella_2	5	3.83	0.83	3.34	5.74	0.054	0.423	0.291	0.476	0.703
Prevotella_2	12	5.81	2.07	1.38	5.47	1.491	0.475	0.886	0.854	0.703
Rikenellaceae_RC9_gut_group	5	3.58	2.80	1.41	3.93	0.496	0.725	0.944	0.398	0.805
Rikenellaceae_RC9_gut_group	12	1.75	4.09	7.30	3.29	1.259	0.157	0.498	0.437	0.805
Actinobacillus	5	4.10	1.30	1.28	2.05	0.004	0.768	0.533	0.896	0.548
Actinobacillus	12	1.68	0.73	3.60	3.45	0.814	0.221	0.462	0.515	0.548
unknown Ruminococcaceae	5	3.21	1.77	1.35	3.25	0.022	0.424	1.000	0.217	0.128
unknown Ruminococcaceae	12	2.88	5.78	2.51	4.70	0.724	0.279	0.158	0.405	0.128
unknown Lachnospiraceae	5	2.62	5.51	2.53^{-}	4.29	0.038	0.622	0.712	0.825	**0.013**
unknown Lachnospiraceae	12	1.08	1.57	0.64^{+}	1.00	0.168	0.157	0.140	0.257	**0.013**
Roseburia	5	0.43	0.07	0.01	0.00	0.002	0.121	0.439	0.392	1.000
Roseburia	12	1.59	0.33	0.08	1.34	0.477	0.884	0.558	0.928	1.000
Prevotellaceae_UCG-003	5	1.44	0.19	0.16	0.25	0.005	0.327	0.606	0.630	0.686
Prevotellaceae_UCG-003	12	0.76	0.27	0.39	0.54	0.115	0.501	0.700	***0.095***	0.686
Romboutsia	5	1.84	2.94	1.55^{F}	1.35	0.006	0.667	0.196	0.440	**0.001**

Romboutsia	12	2.90	3.67	4.28^{E}	2.52	0.426	0.673	0.673	0.791	**0.001**
Streptococcus	5	1.47	0.84^{E}	1.98	0.95	0.026	0.673	0.360	0.732	**0.003**
Streptococcus	12	0.47	0.35^{F}	0.64	0.28	0.100	0.821	0.226	0.637	**0.003**
Prevotellaceae_NK3B31_group	5	1.07	0.75	4.05^{E}	3.41	0.244	**0.016**	1.000	0.107	**0.004**
Prevotellaceae_NK3B31_group	12	0.93	0.54	0.68^{F}	0.44	0.187	0.328	0.965	0.362	**0.004**
Alloprevotella	5	1.05	0.21^{-}	0.27	1.18	0.003	0.845	0.248	0.289	***0.097***
Alloprevotella	12	3.08	1.17^{+}	2.99	3.90	0.859	0.324	0.526	0.651	***0.097***
Lachnoclostridium	5	1.56	1.47	0.78	1.77	0.477	0.242	0.951	0.432	***0.055***
Lachnoclostridium	12	0.88	0.76	0.87	0.61	0.188	0.439	0.573	0.752	***0.055***
Peptostreptococcus	5	0.97	0.23^{E}	0.42	0.21	0.013	0.534	0.354	0.737	***0.001***
Peptostreptococcus	12	0.04	0.00^{F}	0.07	0.04	0.010	0.242	0.866	0.470	***0.001***
Turicibacter	5	0.89	0.30	0.16	0.33	0.014	0.906	1.000	0.870	0.470
Turicibacter	12	0.23	0.21	0.33	0.08	0.066	0.965	0.286	0.745	0.470
Blautia	5	0.90	2.47	0.59	0.37	0.085	0.131	0.470	0.238	*unkonw*
Blautia	12	0.20	0.44	0.29	0.16	0.075	***0.059***	0.958	***0.071***	***0.065***
Parabacteroides	5	1.06	0.71^{E}	1.66	3.09^{E}	0.013	0.902	0.712	0.781	**0.013**
Parabacteroides	12	0.65	0.10^{F}	0.87	0.09^{F}	0.221	0.534	**0.038**	0.193	**0.013**
Collinsella	5	0.34	0.20	0.16	0.22	0.022	0.749	0.808	0.930	0.673
Collinsella	12	0.05	0.03	0.00	0.01	0.012	0.480	1.000	0.670	0.673
Holdemanella	5	0.48	0.11	0.09	0.08	0.068	0.253	0.199	0.181	0.522
Holdemanella	12	0.12	0.29	0.03	0.00	0.078	***0.083***	1.000	0.284	0.522
Veillonella	5	0.74^{+}	0.12	0.34^{E}	0.25^{E}	0.011	0.597	0.131	0.220	**0.025**
Veillonella	12	0.05^{-}	0.04	0.10^{aF}	0.02^{bF}	0.017	0.796	***0.071***	0.193	**0.025**
Ruminococcus_2	5	0.81	0.85	0.51	0.55	0.035	1.000	0.398	0.669	0.351
Ruminococcus_2	12	1.01	0.68	0.27	0.04	0.184	0.131	0.100	0.146	0.351
unknown Pasteurellaceae	5	0.39	0.04	0.10	0.09	0.009	0.817	***0.059***	0.291	0.775
unknown Pasteurellaceae	12	0.05	0.03	0.11	0.08	0.020	**0.006**	0.201	**0.034**	0.775
Escherichia/Shigella	5	0.62	0.18	0.97	0.89	0.018	0.174	0.762	0.174	0.940
Escherichia/Shigella	12	0.70	0.25	0.46	0.15	0.132	0.922	**0.033**	0.113	0.940
Ruminococcaceae_UCG-002	5	0.60	1.27	0.63	0.97	0.007	0.929	0.270	0.715	***0.059***
Ruminococcaceae_UCG-002	12	2.26	2.63	2.54	2.60	0.480	1.000	0.622	0.961	***0.059***

Ruminococcaceae_NK4A214_group	5	0.54	0.53	0.41	0.69	0.000	0.974	0.510	0.891	**0.029**
Ruminococcaceae_NK4A214_group	12	1.15	1.21	0.88	0.93	0.166	0.460	0.806	0.871	**0.029**
Alistipes	5	0.20	0.03	0.09^{a}	0.00^{b}	0.010	0.221	0.121	0.127	0.145
Alistipes	12	0.00^{x}	0.02	0.18^{z}	0.00	0.038	*0.086*	0.796	0.251	0.145
Tyzzerella	5	0.56^{E}	0.22	0.22	0.75^{E}	0.014	0.673	0.439	0.229	**0.048**
Tyzzerella	12	0.16^{F}	0.31	0.10	0.14^{F}	0.042	0.102	**0.043**	0.117	**0.048**
Family_XIII_AD3011_group	5	0.55	0.21^{F}	0.35	0.38^{F}	0.025	0.498	0.806	0.775	**0.003**
Family_XIII_AD3011_group	12	0.68	1.36^{E}	0.92	0.93^{E}	0.179	0.792	***0.065***	0.332	**0.003**
Lachnospiraceae_UCG-004	5	0.08	0.04	0.00	0.04	0.825	0.643	0.143	0.213	0.484
Lachnospiraceae_UCG-004	12	0.16	0.14	0.11	0.14	0.025	**0.050**	1.000	0.250	0.484
Clostridium_sensu_stricto_2	5	0.43	0.51	2.27	0.79	0.070	0.758	0.712	0.957	0.429
Clostridium_sensu_stricto_2	12	0.11	0.08	0.39	0.08	0.079	0.409	0.327	0.568	0.429
Terrisporobacter	5	0.33	0.45	0.22	0.18	0.004	0.184	0.545	0.574	***0.088***
Terrisporobacter	12	0.43	0.85	0.50	0.28	0.140	0.597	0.762	0.946	***0.088***
Prevotella	5	0.06	0.41	0.01	0.09	0.001	0.827	1.000	0.986	0.606
Prevotella	12	0.12	0.01	0.04	0.10	0.026	1.000	0.881	0.761	0.606
unknown Lactobacillales	5	0.06	0.13	0.12	0.15	0.009	0.808	0.610	0.681	0.165
unknown Lactobacillales	12	0.07	0.38	0.23	0.19	0.064	0.565	0.338	0.327	0.165
Epulopiscium	5	0.17	0.27	0.17	0.09	0.014	0.630	0.172	0.580	0.344
Epulopiscium	12	0.05	0.08	0.13	0.14	0.023	0.248	0.630	0.674	0.344
unknown Clostridiaceae_1	5	0.17	0.27	0.46^{E}	0.30	0.003	1.000	0.753	0.692	0.374
unknown Clostridiaceae_1	12	0.07	0.14	0.11^{F}	0.27	0.039	**0.045**	0.144	0.130	0.374
Intestinimonas	5	0.29	0.31	0.11	0.54	0.028	1.000	0.105	0.364	0.353
Intestinimonas	12	0.51	0.47	0.35	0.31	0.064	0.295	0.758	0.740	0.353
Prevotella_9	5	0.05	0.16	0.11	0.18	0.003	0.643	**0.050**	0.232	**0.005**
Prevotella_9	12	0.09	0.04	0.03	0.03	0.023	0.670	0.754	0.862	**0.005**
Ruminococcaceae_UCG-005	5	0.24^{F}	0.19^{F}	0.17^{F}	0.14^{-}	0.003	0.271	***0.053***	0.177	**0.000**
Ruminococcaceae_UCG-005	12	2.11^{E}	3.10^{E}	1.76^{E}	1.91^{+}	0.441	0.178	0.622	0.579	**0.000**
unknown Family_XIII	5	0.14	0.06^{+}	0.04	0.12^{+}	0.006	0.728	0.643	0.174	**0.026**
unknown Family_XIII	12	0.00	0.01^{-}	0.01	0.01^{-}	0.003	0.564	***0.083***	0.284	**0.026**
Dorea	5	0.09	0.39	0.22	0.43	0.057	0.560	0.234	0.634	0.150

Dorea	12	0.57	1.07^{c}	0.42	0.21^{d}	0.112	***0.065***	0.439	0.148	0.150
Christensenellaceae_R-7_group	5	0.22^{F}	0.20^{F}	0.30	0.25	0.081	0.360	0.570	0.747	**0.000**
Christensenellaceae_R-7_group	12	1.78^{E}	2.74^{E}	1.39	1.91	0.334	0.481	0.291	0.600	**0.000**
Eisenbergiella	5	0.25^{E}	0.40^{E}	0.40	0.16	0.109	0.776	0.118	0.249	***0.054***
Eisenbergiella	12	0.04^{F}	0.01^{F}	0.11	0.01	0.026	0.827	***0.064***	0.312	***0.054***
p-1088-a5_gut_group	5	0.08	0.07	0.08^{-}	0.05	0.009	0.101	0.564	0.256	0.317
p-1088-a5_gut_group	12	0.52	0.42	0.35^{+}	0.19	0.117	0.131	0.859	0.396	0.317
Ruminococcaceae_UCG-010	5	0.19	0.15	0.10	0.22	0.032	0.568	0.620	0.824	0.465
Ruminococcaceae_UCG-010	12	0.14	0.21	0.14	0.18	0.034	0.762	0.650	0.701	0.465
Butyricimonas	5	0.22	0.40^{E}	0.13	0.15	0.050	0.380	0.290	0.293	**0.023**
Butyricimonas	12	0.09	0.08^{F}	0.66	0.06	0.154	0.462	**0.022**	***0.090***	**0.023**
Negativibacillus	5	0.04	0.08	0.01	0.09	0.021	0.917	0.794	0.938	1.000
Negativibacillus	12	0.00	0.02	0.00	0.00	0.005	n.a	n.a	n.a	1.000
Ruminiclostridium_9	5	0.20	0.21	0.23	0.33	0.012	0.389	0.295	0.623	**0.045**
Ruminiclostridium_9	12	0.76	0.77	0.27	0.27	0.114	**0.014**	0.573	***0.081***	**0.045**
Phascolarctobacterium	5	0.16	0.27	0.05	0.17	0.023	0.131	0.305	0.102	***0.072***
Phascolarctobacterium	12	0.72	0.79	0.80	0.60	0.251	0.124	0.498	0.245	***0.072***
Oscillospira	5	0.15^{F}	0.20	0.06^{F}	0.16	0.011	0.965	0.859	0.997	**0.017**
Oscillospira	12	0.57^{E}	0.37	0.56^{E}	0.38	0.102	0.375	0.429	0.680	**0.017**
Ruminococcaceae_UCG-004	5	0.18	0.17	0.13	0.20	0.010	0.970	0.821	0.995	**0.006**
Ruminococcaceae_UCG-004	12	0.57	0.43	0.38	0.24	0.094	***0.072***	0.806	0.287	**0.006**
Eubacterium	5	0.05	0.01	0.00	0.01	0.013	0.480	1.000	0.741	0.685
Eubacterium	12	0.04	0.01	0.02	0.01	0.009	1.000	0.480	0.733	0.685
Hungatella	5	0.13	0.27	0.07	0.11	0.019	0.841	0.482	0.893	0.671
Hungatella	12	0.01	0.00	0.20	0.02	0.051	0.655	0.655	0.861	0.671
unknown Paludibacteraceae	5	0.02	0.00	0.02	0.00	0.007	0.221	n.a	0.221	0.197
unknown Paludibacteraceae	12	0.03	0.09	0.04	0.03	0.022	0.513	0.827	0.934	0.197
GCA-900066225	5	0.14^{+}	0.12^{E}	0.15^{+}	0.09^{+}	0.031	0.700	0.650	0.949	***0.051***
GCA-900066225	12	0.02^{-}	0.04^{F}	0.01^{-}	0.00^{-}	0.009	**0.050**	0.827	0.232	***0.051***
Marvinbryantia	5	0.09	0.22	0.14	0.06	0.113	0.346	1.000	0.809	0.610
Marvinbryantia	12	0.06	1.24	0.11	0.07	0.207	0.290	0.149	0.209	0.610

Sutterella	5	0.05	0.01	0.01	0.05	0.002	0.831	0.286	0.187	0.597
Sutterella	12	0.11	0.02^{x}	0.01^{b}	0.07^{az}	0.025	0.917	0.465	0.786	0.597
Fournierella	5	0.04	0.01	0.01	0.04	0.009	1.000	0.624	***0.087***	n.a
Fournierella	12	0.00	0.00	0.00	0.00	0.000	n.a	n.a	n.a	n.a
Acetitomaculum	5	0.00	0.00	0.00	0.00	0.001	0.221	n.a	0.221	0.157
Acetitomaculum	12	0.00	0.01	0.00	0.00	0.002	1.000	0.121	0.392	0.157
Actinomyces	5	0.05	0.01	0.01	0.01	0.007	**0.025**	***0.083***	***0.090***	1.000
Actinomyces	12	0.01	0.00	0.01	0.00	0.003	1.000	0.221	0.368	1.000
Anaerotruncus	5	0.01	0.00	0.00	0.00	0.003	0.317	0.317	0.317	0.355
Anaerotruncus	12	0.00	0.01	0.00	0.01	0.003	0.121	0.655	0.259	0.355
Asteroleplasma	5	0.01	0.00	0.00	0.00	0.003	0.221	1.000	0.368	0.289
Asteroleplasma	12	0.01	0.00	0.00	0.00	0.004	0.121	1.000	0.392	0.289
Bilophila	5	0.00	0.00	0.00	0.00	0.000	n.a	n.a	n.a	n.a
Bilophila	12	0.02	0.00	0.06	0.01	0.010	0.881	0.317	0.692	n.a
Butyricicoccus	5	0.06	0.01	0.02	0.03	0.000	0.277	0.796	0.669	0.217
Butyricicoccus	12	0.02	0.05	0.04	0.05	0.014	0.200	0.167	0.359	0.217
CAG-873	5	0.00	0.00	0.04	0.00	0.005	0.157	0.157	0.157	n.a
CAG-873	12	0.00	0.00	0.00	0.00	0.000	n.a	n.a	n.a	n.a
Campylobacter	5	0.00	0.00	0.00	0.00	0.001	0.317	n.a	0.317	0.117
Campylobacter	12	0.07	0.02	0.63	0.13	0.163	0.386	0.881	0.614	0.117
Candidatus_Soleaferrea	5	0.01^{-}	0.00^{-}	0.00^{F}	0.03	0.000	1.000	1.000	1.000	**0.050**
Candidatus_Soleaferrea	12	0.08^{+}	0.12^{+}	0.07^{E}	0.06	0.021	***0.083***	0.643	0.184	**0.050**
Caproiciproducens	5	0.00	0.00	0.00	0.01	0.002	0.180	0.121	0.259	0.480
Caproiciproducens	12	0.00	0.00	0.00	0.00	0.001	n.a	n.a	n.a	0.480
Clostridium_sensu_stricto_13	5	0.00	0.02	0.02	0.04	0.027	1.000	0.355	0.565	0.423
Clostridium_sensu_stricto_13	12	0.00	0.01	0.01	0.02	0.004	0.643	0.275	0.463	0.423
Clostridium_sensu_stricto_4	5	0.03	0.08	0.02^{F}	0.00	0.163	0.739	0.456	0.373	***0.061***
Clostridium_sensu_stricto_4	12	0.08	0.16	$0.16^{E\#}$	0.08^{*}	0.027	0.563	0.142	0.287	***0.061***
Coprococcus_1	5	0.00	0.00	0.00	0.01	0.002	n.a	n.a	n.a	0.770
Coprococcus_1	12	0.02	0.10	0.01	0.00	0.025	1.000	0.157	0.344	0.770
Coprococcus_3	5	0.02	0.07	0.06	0.01	0.005	0.302	0.624	0.416	0.428

Coprococcus_3	12	0.08	0.14	0.09	0.06	0.023	0.958	0.664	0.487	0.428
Corynebacterium_1	5	0.00	0.00	0.00	0.00	0.000	n.a	n.a	n.a	n.a
Corynebacterium_1	12	0.03	0.06	0.03	0.07	0.022	0.724	***0.053***	0.258	n.a
Defluviitaleaceae_UCG-011	5	0.00	0.00	0.03	0.00	0.007	0.317	0.317	0.317	0.739
Defluviitaleaceae_UCG-011	12	0.00^{x}	0.00	0.02^{z}	0.01	0.004	***0.064***	0.513	0.117	0.739
Denitrobacterium	5	0.03	0.02	0.03	0.04	0.002	0.670	0.394	0.839	1.000
Denitrobacterium	12	0.05	0.06	0.03	0.02	0.009	0.157	0.908	0.418	1.000
Desulfovibrio	5	0.11	0.08	0.08	0.13	0.006	0.510	0.818	0.336	0.650
Desulfovibrio	12	0.13	0.13	0.21	0.07	0.033	0.295	0.498	0.582	0.650
dgA-11_gut_group	5	0.04	0.01	0.02	0.06	0.009	0.462	1.000	0.839	0.676
dgA-11_gut_group	12	0.05	0.10	0.07	0.02	0.018	**0.045**	0.257	0.219	0.676
Enterococcus	5	0.01	0.00	0.00	0.00	0.002	0.180	0.121	0.259	0.157
Enterococcus	12	0.01	0.03	0.00	0.01	0.009	0.221	1.000	0.368	0.157
Erysipelotrichaceae_UCG-004	5	0.01	0.00	0.00	0.10	0.027	0.439	0.180	0.407	0.201
Erysipelotrichaceae_UCG-004	12	0.29	0.00	0.01	0.02	0.054	***0.064***	0.355	0.180	0.201
Faecalibacterium	5	0.01	0.06	0.00^{*}	$0.05^{\#}$	0.015	0.827	0.770	0.953	0.423
Faecalibacterium	12	0.02	0.05	0.00	0.01	0.009	0.355	0.643	0.801	0.423
Family_XIII_UCG-001	5	0.02	0.01	0.00	0.01	0.022	0.796	0.142	0.261	1.000
Family_XIII_UCG-001	12	0.02	0.01	0.01	0.01	0.003	0.305	0.394	0.651	1.000
Flavonifractor	5	0.09	0.05	0.04	0.09	0.002	0.283	0.923	0.738	n.a
Flavonifractor	12	0.00	0.00	0.00	0.00	0.000	n.a	n.a	n.a	n.a
GCA-900066575	5	0.00	0.01	0.00	0.01	0.004	0.221	0.221	0.368	0.926
GCA-900066575	12	0.15^{z}	0.02	0.01^{x}	0.01	0.031	***0.071***	0.462	0.348	0.926
H1	5	0.00	0.00	0.00	0.00	0.000	n.a	n.a	n.a	n.a
H1	12	0.09	0.04	0.04	0.01	0.027	***0.083***	0.564	0.284	n.a
Helicobacter	5	0.00	0.00	0.02	0.08	0.019	n.a	1.000	1.000	0.624
Helicobacter	12	0.01	0.01	0.00	0.02	0.006	1.000	*0.083*	0.284	0.624
horsej-a03	5	0.00	0.01	0.02	0.03	0.006	0.165	1.000	0.300	0.317
horsej-a03	12	0.00	0.00	0.00	0.00	0.001	n.a	n.a	n.a	0.317
Howardella	5	0.04^{c}	0.06^{E}	0.14^{adE}	0.04^{b}	0.004	0.270	**0.021**	*0.097*	**0.039**
Howardella	12	0.01	0.01^{F}	0.02^{F}	0.02	0.005	0.569	0.754	0.753	**0.039**

Hydrogenoanaerobacterium	5	0.09^{E}	0.05^{+}	0.06	0.07^{E}	0.056	0.722	0.135	0.386	**0.013**
Hydrogenoanaerobacterium	12	0.01^{F}	0.02^{-}	0.03	0.00^{F}	0.005	0.465	0.732	0.619	**0.013**
Intestinibacter	5	0.03	0.06	0.02	0.00	0.001	0.480	0.564	0.497	0.752
Intestinibacter	12	0.08	0.08	0.10	0.09	0.028	0.685	0.685	0.444	0.752
Lachnospiraceae_FCS020_group	5	0.00	0.01	0.01	0.01	0.004	0.121	0.180	0.259	***0.086***
Lachnospiraceae_FCS020_group	12	0.04	0.04	0.00	0.01	0.011	0.157	0.564	0.368	***0.086***
Lachnospiraceae_NC2004_group	5	0.01	0.02	0.00^{-*}	$0.05^{\#}$	0.005	1.000	0.480	0.670	0.549
Lachnospiraceae_NC2004_group	12	0.06	0.02	0.10^{+}	0.03	0.018	0.806	0.302	0.311	0.549
Lachnospiraceae_UCG-010	5	0.00	0.09	0.00	0.02	0.009	0.513	0.355	0.543	0.366
Lachnospiraceae_UCG-010	12	0.04	0.05	0.09	0.10	0.022	0.273	0.465	0.638	0.366
Mannheimia	5	0.03	0.00	0.00	0.00	0.006	n.a	1.000	1.000	0.275
Mannheimia	12	0.02	0.00	0.08	0.00	0.020	1.000	n.a	1.000	0.275
Mogibacterium	5	0.00^{F}	0.01	0.00^{-}	0.03	0.004	1.000	n.a	1.000	***0.089***
Mogibacterium	12	0.08^{E}	0.08	0.12^{+}	0.05	0.025	0.391	0.391	0.139	***0.089***
Oscillibacter	5	0.10	0.07	0.01	0.21	0.001	0.465	0.262	0.629	0.963
Oscillibacter	12	0.03	0.13	0.11	0.09	0.024	0.745	0.874	0.851	0.963
Peptococcus	5	0.00	0.00	0.00	0.01	0.002	0.248	0.480	0.223	**0.014**
Peptococcus	12	0.03	0.04	0.00	0.00	0.009	n.a	0.439	0.439	**0.014**
Prevotellaceae_UCG-001	5	0.00	0.00	0.00	0.00	0.000	n.a	n.a	n.a	n.a
Prevotellaceae_UCG-001	12	0.02	0.18	0.00	0.02	0.045	0.739	0.248	0.472	n.a
Prevotellaceae_UCG-004	5	0.06	0.01	0.00	0.22	0.004	0.439	0.724	0.481	0.390
Prevotellaceae_UCG-004	12	0.06	0.09	0.21	0.12	0.052	0.584	0.571	0.696	0.390
Pseudoflavonifractor	5	0.01	0.03	0.01	0.00	0.001	0.245	0.480	0.508	1.000
Pseudoflavonifractor	12	0.03	0.01	0.05	0.01	0.013	0.881	0.881	0.590	1.000
Pygmaiobacter	5	0.00	0.01	0.01	0.01	0.004	1.000	0.248	0.301	0.770
Pygmaiobacter	12	0.00	0.00	0.00	0.00	0.001	n.a	n.a	n.a	0.770
Rothia	5	0.03	0.05	0.02^{F}	0.07	0.103	0.796	***0.071***	0.338	0.138
Rothia	12	0.13	0.06	$0.10^{E\#}$	0.05^{*}	0.021	0.691	***0.076***	0.227	0.138
Ruminiclostridium_5	5	0.02	0.02	0.01	0.01	0.005	***0.088***	0.917	0.320	n.a
Ruminiclostridium_5	12	0.00	0.00	0.00	0.00	0.000	n.a	n.a	n.a	n.a
Ruminococcaceae_UCG-003	5	0.02	0.00	0.00	0.01	0.005	***0.083***	0.248	0.223	0.327

Ruminococcaceae_UCG-003	12	0.01	0.00	0.00	0.01	0.002	0.121	0.121	0.121	0.327
Ruminococcaceae_UCG-009	5	0.03	0.01	0.00	0.04	0.333	0.157	0.248	0.344	0.114
Ruminococcaceae_UCG-009	12	0.04	0.02	0.02	0.02	0.006	0.935	0.109	0.394	0.114
Ruminococcaceae_UCG-013	5	0.01	0.02	0.01	0.00	0.001	1.000	0.480	0.634	0.405
Ruminococcaceae_UCG-013	12	0.03	0.03	0.02	0.03	0.007	0.199	0.668	0.577	0.405
Ruminococcaceae_UCG-014	5	0.00	0.00	0.00	0.00	0.001	n.a	n.a	n.a	0.127
Ruminococcaceae_UCG-014	12	0.01	0.06	0.02	0.07	0.019	0.121	0.289	0.277	0.127
Ruminococcus_1	5	0.00	0.00	0.00	0.00	0.000	n.a	n.a	n.a	n.a
Ruminococcus_1	12	0.04	0.03	0.02	0.02	0.007	0.167	0.570	0.517	n.a
Sarcina	5	0.02	0.10	0.00	0.07	0.026	0.564	0.480	0.497	*0.053*
Sarcina	12	0.00	0.01	0.00	0.00	0.002	0.317	0.317	0.317	*0.053*
Sphaerochaeta	5	0.03	0.02	0.02	0.13	0.000	***0.083***	0.297	0.160	0.907
Sphaerochaeta	12	0.53	0.08	0.67	0.08	0.185	0.700	0.149	0.490	0.907
Staphylococcus	5	0.00	0.01	0.01	0.00	0.002	0.221	0.221	0.221	0.513
Staphylococcus	12	0.00	0.00	0.02	0.00	0.002	n.a	n.a	n.a	0.513
Subdoligranulum	5	0.03	0.28	0.01	0.02	0.045	0.327	0.462	0.523	0.288
Subdoligranulum	12	0.08	0.96	0.30	0.02	0.241	0.251	0.201	0.186	0.288
Treponema_2	5	0.00^{-}	0.00^{-}	0.00^{-}	0.00^{-}	0.003	n.a	0.317	0.317	**0.027**
Treponema_2	12	0.26^{+}	0.21^{+}	0.14^{+}	0.17^{+}	0.081	0.568	0.391	0.793	**0.027**
UBA1819	5	0.10	0.05	0.13	0.08	0.010	0.624	0.414	0.666	0.633
UBA1819	12	0.04	0.08	0.04	0.00	0.022	0.386	1.000	0.250	0.633
unknown Atopobiaceae	5	0.01	0.01	0.00	0.00	0.003	0.564	0.248	0.532	0.327
unknown Atopobiaceae	12	0.00	0.00	0.00	0.00	0.001	1.000	0.121	0.392	0.327
unknown Bacteroidales	5	0.03	0.04	0.02	0.06	0.002	0.917	0.569	0.936	0.329
unknown Bacteroidales	12	0.01	0.02	0.02	0.01	0.006	0.513	0.827	0.836	0.329
unknown Bradymonadales	5	0.00^{-}	1.12	0.78	0.01	0.095	0.289	1.000	0.276	**0.021**
unknown Bradymonadales	12	0.04^{+}	0.19	0.04	0.01	0.032	0.540	1.000	0.387	**0.021**
unknown Carnobacteriaceae	5	0.00	0.20^{+}	0.26^{+}	0.14	0.044	0.305	0.569	0.317	***0.086***
unknown Carnobacteriaceae	12	0.00	0.00^{-}	0.01^{-}	0.02	0.005	n.a	0.317	0.317	***0.086***
unknown Clostridiales	5	0.05	0.09^{-}	0.04^{-}	0.03^{-}	0.008	0.203	0.814	0.625	**0.004**
unknown Clostridiales	12	0.06	0.17^{+}	0.11^{+}	0.13^{+}	0.021	0.825	0.248	0.627	**0.004**

unknown Clostridiales_vadinBB60_group	5	0.01^{F}	0.11^{F}	0.66	0.19	0.054	***0.088***	0.465	0.197	*0.083*
unknown Clostridiales_vadinBB60_group	12	3.51^{E}	0.70^{E}	1.47	0.40	0.544	1.000	**0.049**	0.212	*0.083*
unknown Desulfovibrionaceae	5	0.00	0.00	0.00	0.00	0.001	n.a	n.a	n.a	0.143
unknown Desulfovibrionaceae	12	0.01	0.01	0.01	0.00	0.003	0.248	0.480	0.497	0.143
unknown Erysipelotrichaceae	5	0.03^{-}	0.24	0.01^{b}	0.20^{a+}	0.020	0.462	**0.004**	**0.020**	0.719
unknown Erysipelotrichaceae	12	0.24^{a+}	0.02^{b}	0.04	0.03^{-}	0.040	0.626	**0.003**	**0.016**	0.719
unknown F082	5	0.00	0.00	0.00	0.00	0.000	n.a	n.a	n.a	n.a
unknown F082	12	1.93	1.95	3.08	1.59	0.825	0.715	0.850	0.892	n.a
unknown Firmicutes	5	0.08^{-}	0.12	0.17	0.08	0.040	0.825	0.214	0.196	**0.048**
unknown Firmicutes	12	0.17^{+}	0.22	0.15	0.25	0.034	0.744	0.462	0.753	**0.048**
unknown Fusobacteriaceae	5	0.06	0.06	0.09	0.04	0.011	0.749	0.406	0.350	0.781
unknown Fusobacteriaceae	12	0.02	0.00	0.04	0.03	0.012	1.000	1.000	1.000	0.781
unknown Mollicutes_RF39	5	0.00	0.00	0.00	0.00	0.000	n.a	n.a	n.a	n.a
unknown Mollicutes_RF39	12	0.36	0.10	0.01	0.13	0.095	0.564	0.456	0.708	n.a
unknown p-2534-18B5_gut_group	5	0.00	0.00	0.16	0.00	0.003	n.a	n.a	n.a	0.540
unknown p-2534-18B5_gut_group	12	1.62	1.80	0.30	1.61	0.496	0.600	0.791	0.931	0.540
unknown Peptococcaceae	5	0.02	0.01	0.01	0.22	0.025	1.000	0.602	0.929	0.572
unknown Peptococcaceae	12	0.01	0.00	0.01	0.01	0.003	0.655	0.180	0.259	0.572
unknown Peptostreptococcaceae	5	0.00	0.01	0.01	0.01	0.020	1.000	0.221	0.368	0.186
unknown Peptostreptococcaceae	12	0.02	0.06	0.03	0.06	0.013	0.361	0.100	0.229	0.186
unknown Prevotellaceae	5	0.04	0.05^{d}	0.06	0.22^{cE}	0.003	**0.022**	0.537	0.120	0.355
unknown Prevotellaceae	12	0.17	0.20	0.01	0.04^{F}	0.050	***0.055***	0.465	0.223	0.355
unknown Streptococcaceae	5	0.00	0.00	0.04	0.01	0.006	0.157	0.564	0.344	0.380
unknown Streptococcaceae	12	0.00	0.00	0.01	0.00	0.003	n.a	n.a	n.a	0.380
unknown vadinBE97	5	0.01	0.00	0.00	0.02	0.005	0.157	0.480	0.180	0.850
unknown vadinBE97	12	0.01	0.00	0.00	0.00	0.002	0.121	n.a	0.121	0.850
unknown WCHB1-41	5	0.00	0.01	0.00	0.00	0.004	n.a	n.a	n.a	0.827
unknown WCHB1-41	12	0.04	0.03	0.19	0.25	0.068	0.699	0.724	0.895	0.827
unknown WPS-2	5	0.00	0.00	0.00	0.00	0.000	n.a	n.a	n.a	n.a
unknown WPS-2	12	0.19	1.48	0.04	0.38	0.333	0.386	0.180	0.423	n.a

Victivallis	5	0.02	0.00	0.00	0.06	0.014	1.000	1.000	1.000	0.643
Victivallis	12	0.00	0.00	0.02	0.00	0.005	0.317	n.a	0.317	0.643

[1]Values are Means of relative abundance, the largest SE is shown; *n* = 5 / group (5, 12 d).

Colon digesta samples were obtained at 2 h after oral administration of milk replacer and Gln or Ala supplement and snap frozen in liquid nitrogen.

[2] Kruskal Wallis Test, asymptotic significance (significant differences are marked in bold, trends in bold and italics)

Asymptotic significance, none of the other fixed effects or their combination were significant $(p < 0.05)$

[a, b]Labeled Means in a row within one BiW group and one age group without a common letter differ, $p < 0.05$ (Mann-Whitney-U-test).

[c,d]Labeled Means in a row within one supplementation group and one age group without a common letter differ, $p < 0.05$ (Mann-Whitney-U-test).

[e,f]Labeled Means in a column within one supplementation group and BiW group without a common letter differ, $p < 0.05$ (Mann-Whitney-U-test).

[x,z] Labeled Means in a row within one BiW group and one age group without a different superscripts differ , $p < 0.1$ (Mann-Whitney-U-test).

[*,#]Labeled Means in a row within one supplementation group and one age group without a common letter differ, $p < 0.05$ (Mann-Whitney-U-test).

[+,-] Labeled Means in a column within one supplementation group and BiW group without a common letter differ, $p < 0.1$ (Mann-Whitney-U-test).

Ala = Alanine; BiW = birthweight; Gln = Glutamine; LBW = low birthweight; NBW = normal bodyweight; n.a = not available; SE = standard error; Supp = supplementation group

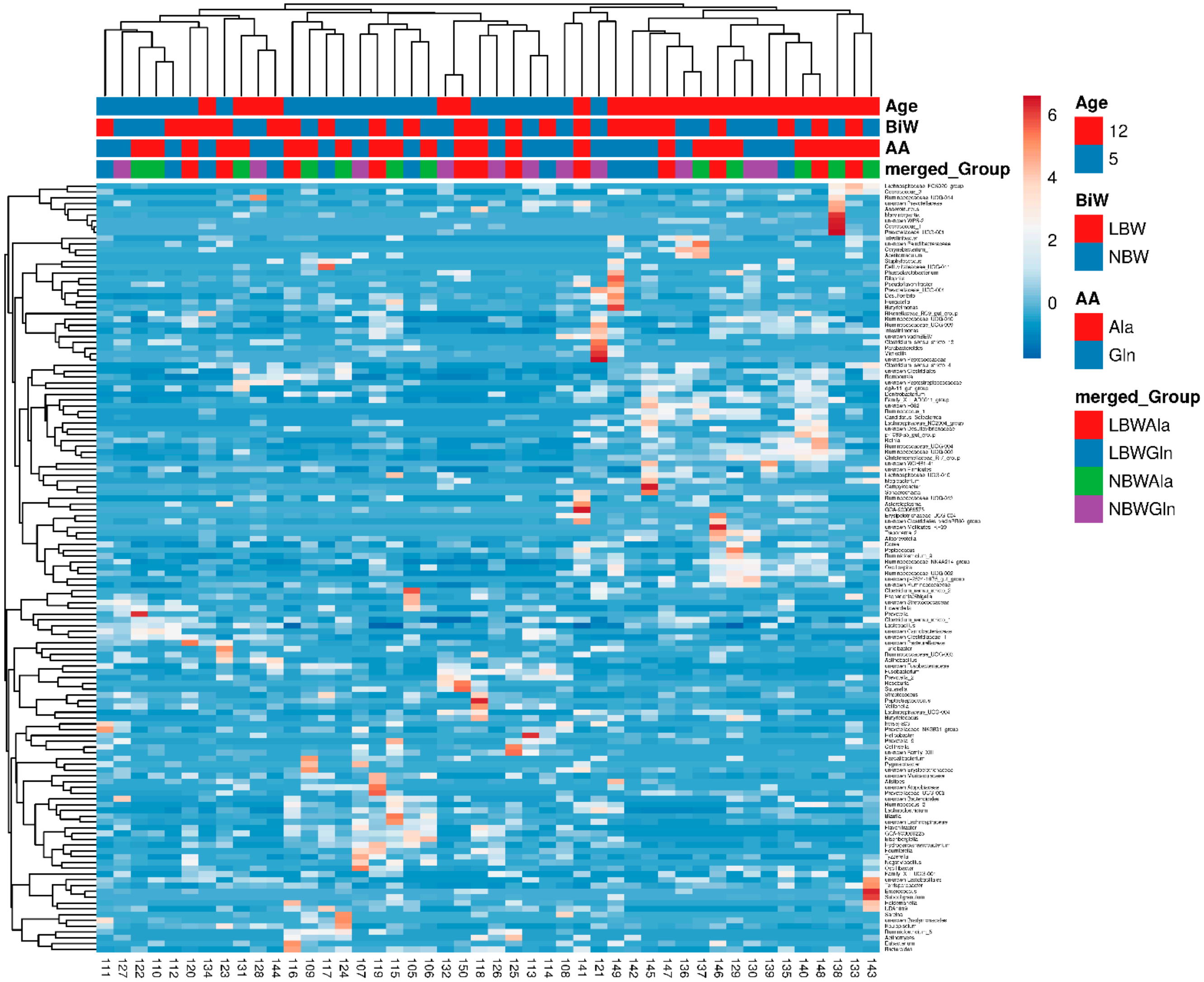

Figure S1. Hierarchical clustering of bacterial genera in colon of male suckling piglets

AA = amino acids; Ala = Alanine; BiW = birthweight; Gln = Glutamine; LBW = low birthweight; NBW = normal birthweight;
